# Golgi Apparatus-Localized Synaptotagmin 2 Is Required for Unconventional Secretion in *Arabidopsis*

**DOI:** 10.1371/journal.pone.0026477

**Published:** 2011-11-28

**Authors:** Haiyan Zhang, Liang Zhang, Bin Gao, Hai Fan, Jingbo Jin, Miguel A. Botella, Liwen Jiang, Jinxing Lin

**Affiliations:** 1 Key Laboratory of Photosynthesis and Molecular Environmental Physiology, Institute of Botany, Chinese Academy of Sciences, Beijing, China; 2 Graduate School of Chinese Academy of Sciences, Beijng, China; 3 College of Life Sciences, Shandong Normal University, Jinan, China; 4 Departamento de Biología Moleculary Bioquímica, Universidad de Málaga, Málaga, Spain; 5 Department of Biology and Molecular Biotechnology Program, The Chinese University of Hong Kong, Shatin, New Territories, Hong Kong, China; Iowa State University, United States of America

## Abstract

**Background:**

Most secretory proteins contain signal peptides that direct their sorting to the ER and secreted via the conventional ER/Golgi transport pathway, while some signal-peptide-lacking proteins have been shown to export through ER/Golgi independent secretory pathways. Hygromycin B is an aminoglycoside antibiotic produced by *Streptomyces hygroscopicus* that is active against both prokaryotic and eukaryotic cells. The hygromycin phosphotransferase (HYG^R^) can phosphorylate and inactivate the hygromycin B, and has been widely used as a positive selective marker in the construction of transgenic plants. However, the localization and trafficking of HYG^R^ in plant cells remain unknown. Synaptotagmins (SYTs) are involved in controlling vesicle endocytosis and exocytosis as calcium sensors in animal cells, while their functions in plant cells are largely unclear.

**Methodology/Principal Findings:**

We found *Arabidopsis* synaptotagmin SYT2 was localized on the Golgi apparatus by immunofluorescence and immunogold labeling. Surprisingly, co-expression of SYT2 and HYG^R^ caused hypersensitivity of the transgenic *Arabidopsis* plants to hygromycin B. HYG^R^, which lacks a signal sequence, was present in the cytoplasm as well as in the extracellular space in *HYG^R^*-*GFP* transgenic *Arabidopsis* plants and its secretion is not sensitive to brefeldin A treatment, suggesting it is not secreted via the conventional secretory pathway. Furthermore, we found that HYG^R^-GFP was truncated at carboxyl terminus of HYG^R^ shortly after its synthesis, and the cells deficient SYT2 failed to efficiently truncate HYG^R^-GFP,resulting in HYG^R^-GFP accumulated in prevacuoles/vacuoles, indicating that SYT2 was involved in HYG^R^-GFP trafficking and secretion.

**Conclusion/Significance:**

These findings reveal for the first time that SYT2 is localized on the Golgi apparatus and regulates HYG^R^-GFP secretion via the unconventional protein transport from the cytosol to the extracelluar matrix in plant cells.

## Introduction

The secretory pathway traditionally contains a number of biochemically distinct inter-related membrane organelles that continuously communicate with each other and exchange materials through membrane trafficking. The classical secretory proteins are often extended at their N-terminus by a ‘leader’ or ‘signal’ sequence of 13–30 hydrophobic amino acids. This directs the nascent protein to co-translate and vectorially transfer across the membrane of the endoplasmic reticulum (ER), and is often cleaved before completion of the transmembrane transport of the protein [1], [2]. Secretory proteins are then transported to the Golgi apparatus and trans-Golgi network where they undergo further glycosylation, and sorting and being packaged into vesicles, respectively. Finally the secretory vesicles are delivered to and fuse with the plasma membrane, resulting in releasing their contents into the extracellular space [3].

However, numerous secretory proteins with normal extracellular functions have been shown to be devoid of functional signal sequences and do not appear substrates for the ER membrane translocation machinery. In addition, the secretion of these proteins is not affected by the presence of brefeldin A, a drug that blocks ER/Golgi-dependent secretory transport [4]–[6]. These observations suggest that alternative secretory mechanisms that are independent of ER/Golgi secretory pathway exist in eukaryotic cells. Secretion of proteins without an N-terminal signal sequence is currently known as the unconventional/non-classical secretory pathway or leaderless secretion. Up to date, several unconventional secretory pathways have been reported for a few biomedically important factors, including proangiogenic mediators such as fibroblast growth factors 2 and inflammatory cytokines such as interleukin 1α and 1β in mammalian cells [5], [7]. Plant secretome revealed that more than half of the total identified proteins were leaderless secretory proteins, which is distinctly higher than in human and yeast secretomes, implying that this unconventional secretory mechanism is common to all eukaryotes and it is more largely used than in other eukaryotes [8]. Furthermore, plants exposed to biotic and abiotic stresses usually significantly contained more leaderless secretory proteins in the extracelluar space than non-stressed plants, suggesting that environmental component might be involved in release of leaderless secretory proteins into the extracelluar space [8]. However, until now, only one leaderless secretory protein, mannitol dehydrogenase (MTD) in celery, has been shown to bypass the ER-Golgi-plasma membrane exocytic pathway for its delivery to the extracellular space by molecular biology and biochemistry approaches [6].

Synaptotagmins (SYTs) constitute a family of membrane-trafficking proteins that are characterized by an N-terminal transmembrane region, a linker of variable size, and two C-terminal C2 domains in tandem [9]. SYTs are reported to play a vital role in neurotransmitter release and insulin exocytosis in mammalian cells [10]–[13].The synaptotagmin family in *Arabidopsis* has five members. SYT1, the only one characterized so far, is ubiquitously expressed and predominantly localized to the plasma membrane [14]. Disruption of *SYT1* function in *Arabidopsis* leads to abiotic stresses hypersensitivity due to a reduced integrity of the plasma membrane [14], [15]. However, the subcellular localization and the functions of other SYTs remain unknown.

Hygromycin B is an aminoglycoside antibiotic produced by *Streptomyces hygroscopicus* that is active against both prokaryotic and eukaryotic cells by inhibiting protein synthesis [16], . It has been reported that hygromycin B acts by interfering with translocation and causes mistranslation [18]. An *Escherichia coli* gene has been identified that confers resistance in transgenic plants against hygromycin B. The resistance gene codes for hygromycin B phosphotransferase (HYG^R^, E.C. 2.7.1.119) that adds phosphate to position 7 of the destomic acid ring of hygromycin B, which results in complete loss of biological activity both *in vitro* and *in vivo* [19]. Although HYG^R^ has been mainly used as a positive selective marker for transgenic cells [20], few studies have examined the subcellular localization and trafficking of HYG^R^ and the putative elements that regulate the tolerance of *HYG^R^*-expressing cells to hygromycin B.

Here, we provided several lines of evidence about localization of *Arabidopsis* synaptotagmin SYT2. More importantly, we found that HYG^R^ is present both in the cytoplasm and the extracelluar space in *HYG^R^*-*GFP*-transgenic plants. The loss of SYT2 caused inhibition of HYG^R^-GFP trafficking. Based on the fact that HYG^R^-GFP lacks a signal sequence and its secretion is not sensitive to brefeldin A treatment, we propose that HYG^R^-GFP is not secreted via the conventional secretory pathway and SYT2 plays an important role in regulating the unconventional protein trafficking from the cytosol to the extracelluar matrix in plant cells.

## Results

### Characterization of *Arabidopsis* SYT2 Protein

**Figure 1:**
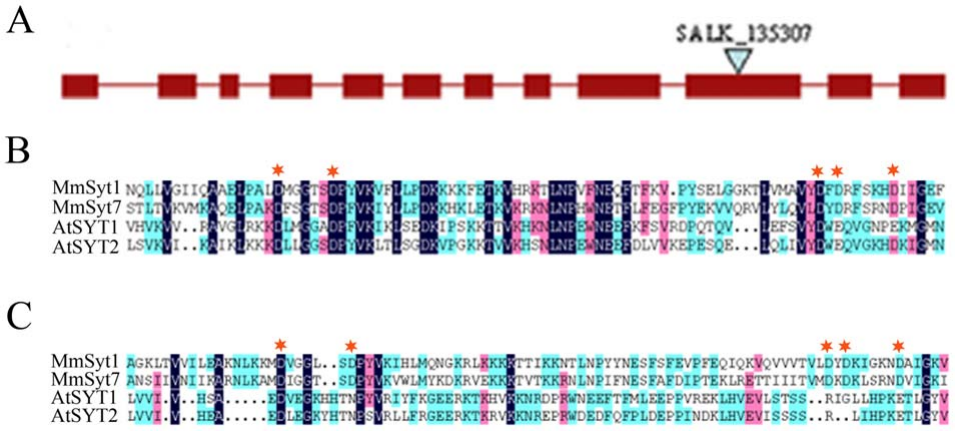
Characteristics of *SYT2* gene in *Arabidopsis thaliana*. Schematic structure of *Arabidopsis* synaptotagmin 2 gene (*SYT2*, *At1g20080*). Solid boxes are exons, lines between the boxes are the introns. The start codon ATG and the stop codon TGA are marked. Triangle indicates the T-DNA insertion site in *syt2-1* (SALK_135307) mutant. (B and C) Amino acid sequence alignment of the C2A (C) and C2B (D) domain of SYT1 and SYT2 in *Arabidopsis* and of mouse Syt1 and Syt2 using the multiple alignment program of Vector NTI Suite 7 (Invitrogen). Asterisks indicate the amino acids involved in calcium binding.

The synaptotagmin 2 gene (*SYT2*, *At1g20080*) is one of five putative *SYTs* in *Arabidopsis*. It comprises 12 exons and 11 introns, based on information available in the *Arabidopsis* Information Resource database (TAIR; http://www.arabidopsis.org/) (Figure 1*A*). Homology analysis using amino acid sequence data showed that SYT1 is the closest relative of SYT2 in *Arabidopsis*, with about 66% amino acid identity between them [15]. Compared to SYT1, all amino acid residues thought to play crucial roles in coordinating calcium (Ca) ions are conserved in the C2A domain of SYT2 (Figure 1B). Unlike SYT1, however, only four putative amino acids of the SYT2 C2B domain are involved in Ca binding (lacking the fourth putative residue) (Figure 1C). According to *SYT1* expression profiles based on microarray expression data obtained from Geneinvestigator (http://www.genevestigator.ethz.ch), *SYT2* is highly expressed in pollen grains, whereas expression in other organs, such as roots or leaves, is detectable but low (Figure S1). A secretory signal peptide was predicted in the SYT2 amino acid sequence but neither a chloroplast transit peptide nor a mitochondrial targeting peptide was identified using the TargetP 1.1 server (http://www.cbs.dtu.dk/services/TargetP/).

### SYT2 does not Colocalize with BFA Compartment

**Figure 2:**
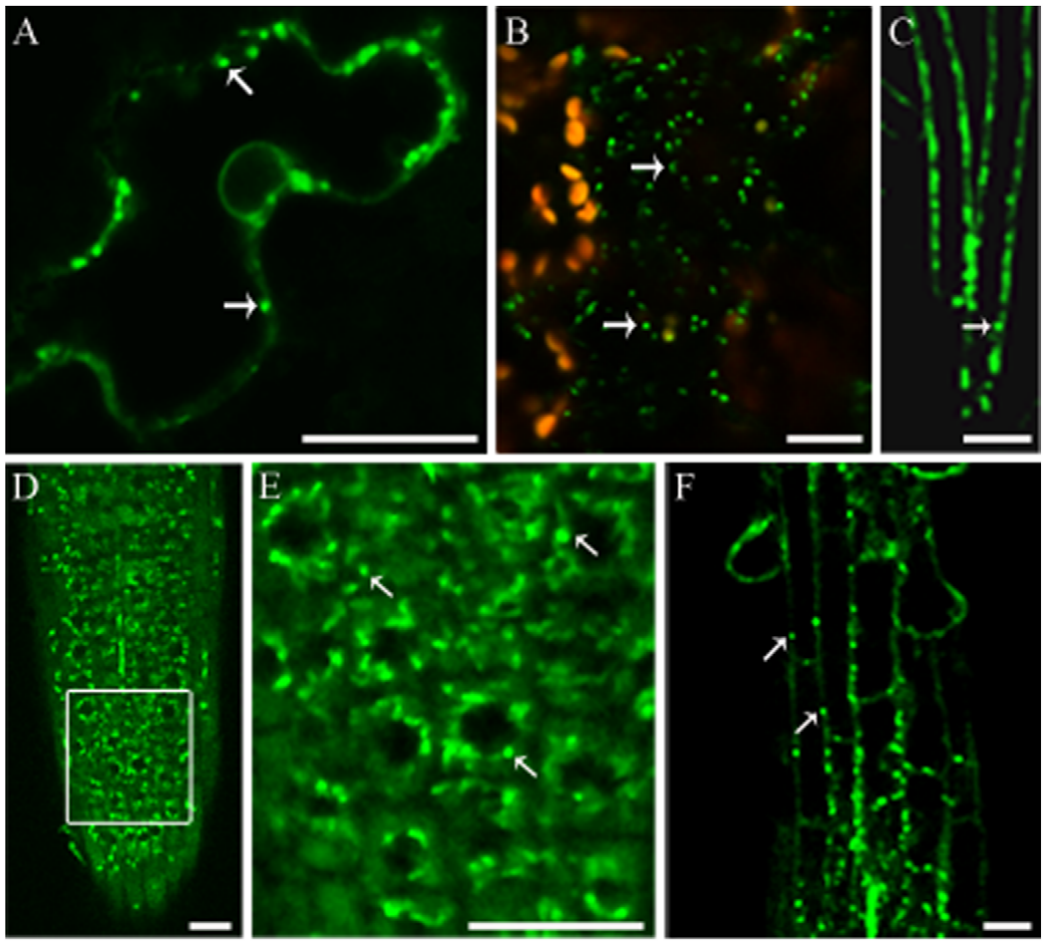
Subcellular localization of SYT2 in *Arabidopsis*. (A and B) Transient expression of SYT2-GFP in leaf epidermis cells of tobacco (A) and *Arabidopsis* (B) shows punctate structures in the cytoplasm. Autofluorescence of chloroplasts appears as golden structures (B). Arrows indicate the punctate structures of SYT2-GFP. Bars = 20 µM. (C–F) Expression of SYT2-GFP in stably transformed root hairs (C) and root tip cells (D–F). (E), High-magnification image of root cells in the inset in (D). Arrows indicate the punctate structures of SYT2-GFP. Bars = 20 µM.

To investigate the subcellular localization of SYT2, we fused the gene encoding green fluorescent protein (GFP) to the *SYT2* gene (C-terminal end of the encoded protein) under the control of the 35S promoter of the cauliflower mosaic virus (*CaMV35S*) and used these constructs to transiently or stably transform *Nicotiana tabacum* and *Arabidopsis*. The resulting fusion protein (SYT2-GFP) was primarily detected in mobile punctate structures in leaf cells of transiently transformed *N. tabacum* and *Arabidopsis* (Figure 2A and 2B). Plant lines stably expressing SYT2-GFP were also analyzed by laser scanning confocal microscope (LSCM) to localize the fusion protein. The fluorescence signals appeared as punctate structures with a dim cytosolic background in root hairs, root meristem cells and elongation cells (Figure 2C to 2F).

**Figure 3:**
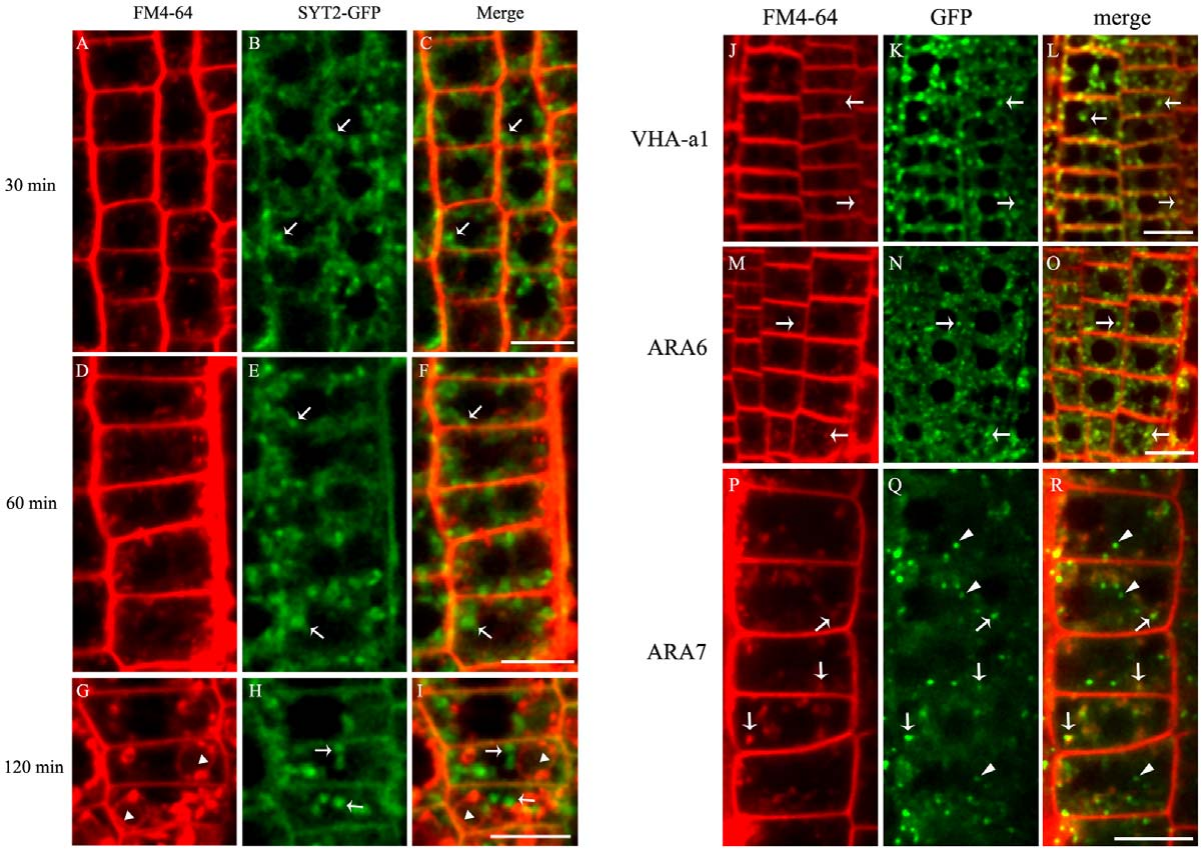
SYT2-GFP does not colocalize with FM4-64-positive compartments. (A–I) Confocal sections of root cells labeled with FM4-64 (red) at room temperature and incubated for 30 min (A–C), 60 min (D–F) and 120 min (G–I). Arrows indicate the punctate structures of SYT2-GFP (green); arrowheads denote vacuolar membranes. Bars = 20 µM. (J–R) Root cells containing VHA-a1-GFP (J–L), ARA6-GFP (M–O) and ARA7-GFP (P–R) were labeled with FM4-64 at room temperature and Confocal sections were taken after a 30-min incubation. Arrows indicate overlapping spots of GFP (green) and FM4-64 (red) fluorescence. Arrowheads indicate that GFP fluorescence did not overlap with that of FM4-64. Bars = 20 µM.

To investigate whether the SYT2-positive structures were of endosomal origin, transgenic SYT2-GFP *Arabidopsis* seedlings were incubated with FM4-64, a fluorescent marker internalized by a clathrin-dependent process and sequentially labels early endosomal, late endosomal, and vacuolar compartments [21]–[24]. As shown in Figure 3, the internalized FM4-64 dye rarely co-localized with SYT2-GFP-containing compartments in root cells even after 2 h of incubation, during which time FM4-64 was detected in vacuolar membranes (Figure 3A to 3I). Co-localization studies were also performed using seedlings expressing VHA-a1-GFP, ARA6-GFP, and ARA7-GFP, all of which have been reported to reside on endosomes and regulate endosomal fusion [21], [23], [25]. Co-localization of FM4-64 with large amounts of VHA-a1-GFP and ARA6-GFP (Figure 3J to 3O) and lesser amounts of ARA7-GFP (Figure 3P to 3R), was detected after 30 min, demonstrating that SYT2-GFP is targeted to a compartment independent of endosomal membranes.

The endosomes in *Arabidopsis* root tips are the main target of the fungal toxin brefeldin A (BFA). This drug inhibits certain ADP ribosylation factor/guanine nucleotide exchange factors (ARF-GEFs) and causes the endocytic tracer FM4-64 to rapidly aggregate throughout vesicle agglomerations known as BFA compartments [26]–[28]. To investigate whether SYT2-GFP was associated with BFA-sensitive endosomes, the transgenic plants were treated with 25 µM BFA. After BFA treatment, the punctate SYT2-GFP structures were almost intact and did not accumulate in BFA compartments (Figure S2A to S2F), while VHA-a1-GFP (early endosome marker) and ARA6-GFP (late endosome marker) perfectly overlapped with and was located at the periphery of the BFA compartments, respectively (Figure S2G to S2L) [23], [29].

To further demonstrate that SYT2-GFP-containing structures are excluded from the late endosomes, we analyzed the effect of wortmannin, which inhibits the biosynthesis of phosphatidylinositol 3- and 4-phosphates as well as phospholipids in plant cells [30]. Exogenous application of wortmannin causes the late endosomes to dilate or form ring-shaped structures, but has no effect on the Golgi apparatus and early endosomes [29], [31]. The morphology of SYT2-GFP structures was not altered and the two markers were almost separated (Figure S3A to S3C). As previously reported for ARA6-GFP and ARA7-GFP, both of which localize on the late endosomes [29], wortmannin caused the formation of ring-shaped structures (Figure S3D to S3I). Furthermore, SYT2 protein did not colocalize with ARA7-GFP by immunofluorescent labelingusing anti-SYT2 and anti-GFP antibodies (Figure S3J to S3L). Taken together, these data demonstrate that SYT2-GFP does not localize on the late endosomes.

### SYT2 is Localized on the Golgi Apparatus

**Figure 4:**
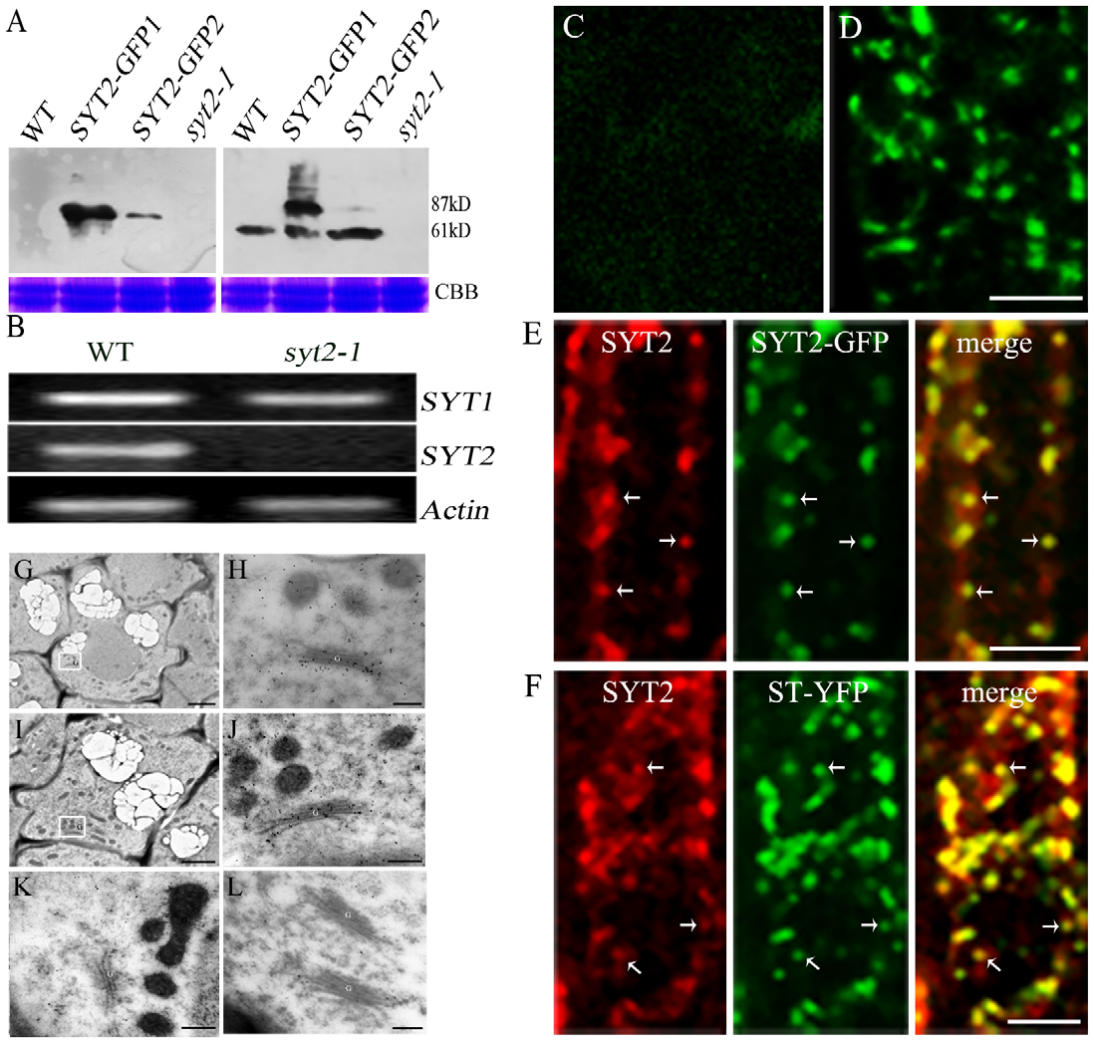
Immunolocalization of SYT2. Bars = 10 µm. (A) Protein gel blot of SYT2 in wild-type (WT), *syt2-1*, and SYT2-GFP overexpressing plants (SYT2-GFP1 and SYT2-GFP2 are different lines). The blots were probed with polyclonal anti-SYT2 (Right) and monoclonal anti-GFP (Left) antibodies to detect the SYT2 and GFP-tagged proteins, respectively. The expected sizes of the proteins are indicated. The bottom images show Coomassie Brilliant Blue staining (CBB) as loading controls. (B) RT-PCR analysis of *SYT1* and *SYT2* in *syt2-1* and wild-type plants. *Actin* served as a control. (C and D) Localization of SYT2 in root cells of wild-type plants. Root tissues of *Arabidopsis* grown on ½ MS solid medium for 3–4 days were prepared for immunolabeling with normal rabbit serum (as a control) (C), or anti-SYT2 antibody as the primary antibody (D) and fluorescein isothiocyanate (FITC)-labeled anti-rabbit IgG as the secondary antibody. (E) Double-labeling with anti-SYT2 and anti-GFP antibodies in root cells of SYT2-GFP-overexpressing seedlings. Anti-SYT2 and anti-GFP antibodies were labeled with tetramethylrhodamine-5-isothiocyanate (TRITC)-labeled anti-rabbit IgG and FITC-labeled anti-rat IgG, respectively. Arrows indicate the overlap of green and red fluorescent signals. (F) Double-labeling with anti-SYT2 and anti-GFP antibodies in root cells containing Golgi marker ST-YFP. Anti-SYT2 and anti-GFP antibodies were used as in (E). Arrows indicate the overlap of green and red fluorescence signals. (G–L) Immuno-gold labeling and electron microscopic observation showed that SYT2 was located on Golgi apparatus in root tip cells of *Arabidopsis*. (G and H) Electron microscopic observation showed that SYT2 was located mainly on Golgi apparatus in root tip cells of wild type plants. (H) High-magnification image of Golgi apparatus in the inset in (G). (I and J) Immuno-gold labeling of Golgi apparatus in root tip cells of *SYT2*-overexpressing plants. (J) High-magnification image of Golgi apparatus in the inset in (I). (K and L) Control section, incubated with the secondary antibody alone, did not show gold particles on Golgi apparatus. G: Golgi apparatus. Bars: 2 µm (G, I,); 50 nm (H, J, K, L).

The punctate structures labeled by SYT2-GFP were insensitive to BFA and wortmannin treatment and did not become labeled with FM4-64, reminiscent of the Golgi apparatus. In addition to its punctate appearance, the Golgi apparatus did not become co-localized with the endocytic tracer FM4-64 or with BFA compartments in *Arabidopsis* root-tip cells [32]–[34]. To determine if SYT2-containing structures were associated with Golgi apparatus, we performed an immunofluorescent study on wild-type and SYT2-GFP-overexpressing plants. Antibodies were generated against the cytoplasmic region (300 aa–535 aa) of SYT2 (anti-SYT2). Affinity purified anti-SYT2 antibodies was analyzed by western blotting of wild type and SYT2-GFP transgenic *Arabidopsis* seedlings, recognizing proteins of 61 KD and 87 kD corresponding to both native SYT2 and recombinant SYT2-GFP respectively (Figure 4A). In order to determine whether the generated antibodies were specific for SYT2 and did not recognize other SYT family members, including the closely related SYT1, a mutant in *SYT2* from the SALK collection (SALK_135307, *syt2-1*) with the T-DNA located in the 9th exon of *At1g20080* was isolated and further analyzed. No mRNA transcripts and proteins were detected in the homozygous *syt2-1* line, despite the fact that *SYT1* was expressed at wild-type levels in the mutant (Figure 4A and 4B). In view of the lower *SYT2* transcripts in vegetative tissues analyzed by microarray ananlysis (Figure S1), it is suggested that SYT2 protein production is probably regulated at the level of translation.

As shown in Figure 4D, SYT2 became immunolocalized into punctate structures were similar to those observed in plants expressing SYT2-GFP. Furthermore, most of the co-localization between SYT2 and SYT2-GFP occurred in transgenic plants over-expressing SYT2-GFP, as confirmed using anti-SYT2 and anti-GFP antibodies (Figure 4E). SYT2 localization was further analyzed by double-immunofluorescent labeling with anti-SYT2 and anti-GFP antibodies in plants expressing ST-YFP, a well described Golgi marker [23], [32]. SYT2 likewise co-localized with ST-YFP (Figure 4F). Immuno-labeling of ultra-thin sections of *Arabidopsis* root cells using anti-SYT2 antibodies showed that gold particles mainly deposited on the Golgi apparatus (Figure 4G to 4L).

### Co-expression of *SYT2* and *HYG^R^* Leads to Hypersensitivity to Hygromycin B

Hygromycin B is an aminoglycoside antibiotic produced by *Streptomyces hygroscopicus* that is active against both prokaryotic and eukaryotic cells [16]. The hygromycin B phosphotransferase (HYG^R^) phosphorylates and inactivates hygromycin B, and has been widely used as a selectable marker in the generation of transgenic plants. To investigate the subcellular localization of SYT2 in plant cells as described above, the binary vector (pCambia1301-SYT2/HYG^R^), which was generated by pCambia1301 from T-DNA containing a SYT2-GFP expression cassette and a hygromycin B-selectable marker, was introduced into *Arabidopsis* seedlings by *Agrobacterium*-mediated transformation. However, we noticed that the positively transgenic plants (named *SYT2/HYG^R^*) grew weakly when selected on 20 µg/mL hygromycin B-containing medium. These plants had low viability or showed slow growth and the apparent loss of apical dominance (inhibition of the primary inflorescence) following the development of two symmetrical axillary buds after their transfer to soil (Figure S4A to S4C). T2 and T3 seedlings exhibited wild-type growth in the absence of hygromycin B. To observe whether SYT2 was associated with uptake of hygromycin B, wild-type and *syt2-1* plants were incubated on ½ Murashige and Skoog (MS) medium containing different concentrations of hygromycin B. As shown in Figure S5, the phenotype of *syt2-1* is not obviously different from that of wild type under hygromycin B treatment, indicating that SYT2 is not probably related with the uptake of hygromycin B in *Arabidopsis*. Therefore, it is presumed that SYT2 contributes to the detoxification of *HYG^R^* in *HYG^R^*-containing plants. To investigate the effect of SYT2 on hygromycin B tolerance, wild-type and *syt2-1* plants were transformed with a *35S-HYG^R^* construct and the transgenic plants were named as *HYG^R^* and *syt2-1/HYG^R^*, respectively.

**Figure 5:**
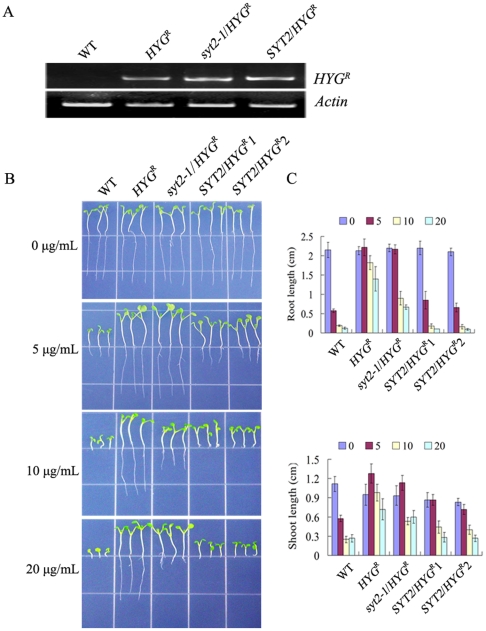
Co-expression of SYT2 and HYG^R^ in *Arabidopsis* induced hypersensitivity to hygromycin B. (A) Reverse transcriptase-PCR (RT-PCR) analysis of *HYG^R^* expression in wild type (WT), *HYG^R^*, *syt2-1/HYG^R^* and *SYT2/HYG^R^* plants. *Actin* was used as a control. (B) Response of wild-type (WT), *HYG^R^*, *syt2-1*/*HYG^R^ and SYT2/HYG^R^* plants to hygromycin B. Seeds were germinated on ½ MS medium supplemented with indicated concentrations of hygromycin B and grew for 7 days before images were taken. *SYT2/HYG^R^1* and *SYT2/HYG^R^2* were different lines that were simultaneously transformed with *SYT2-GFP* and *HYG^R^* genes. (C) Measurement of the length of roots and shoots for seedlings treated as described for (B). Values are the means ± SD of 30–40 seedlings from three independent experiments.

We analyzed the expression of the *HYG^R^* gene in these transgenic lines using semi-quantitative RT-PCR (Figure 5A). The expression level of *HYG^R^* gene was similar in the selected lines. We further investigated the growth of these lines on ½ MS medium agar plates with different concentrations of hygromycin B. The growth of the primary roots and hypocotyls of *SYT2/HYG^R^* seedlings was greatly inhibited even on the medium containing as low as 5 µg/mL hygromycin B (Figure 5B). The roots of *SYT2/HYG^R^* seedlings were 30.1%, 9.3% and 7.1% of that of *HYG^R^* seedlings in the presence of 5, 10 and 20 µg/mL of hygromycin B, respectively (Figure 5C). Microscopic observation also revealed that the root hairs and roots of *SYT2/HYG^R^* seedlings were greatly shortened (Figure S4D to S4L), suggesting that a reduced function of HYG^R^ caused by the over-expression of *SYT2*.

### HYG^R^-GFP is Exported via an Unconventional Secretory Pathway

**Figure 6:**
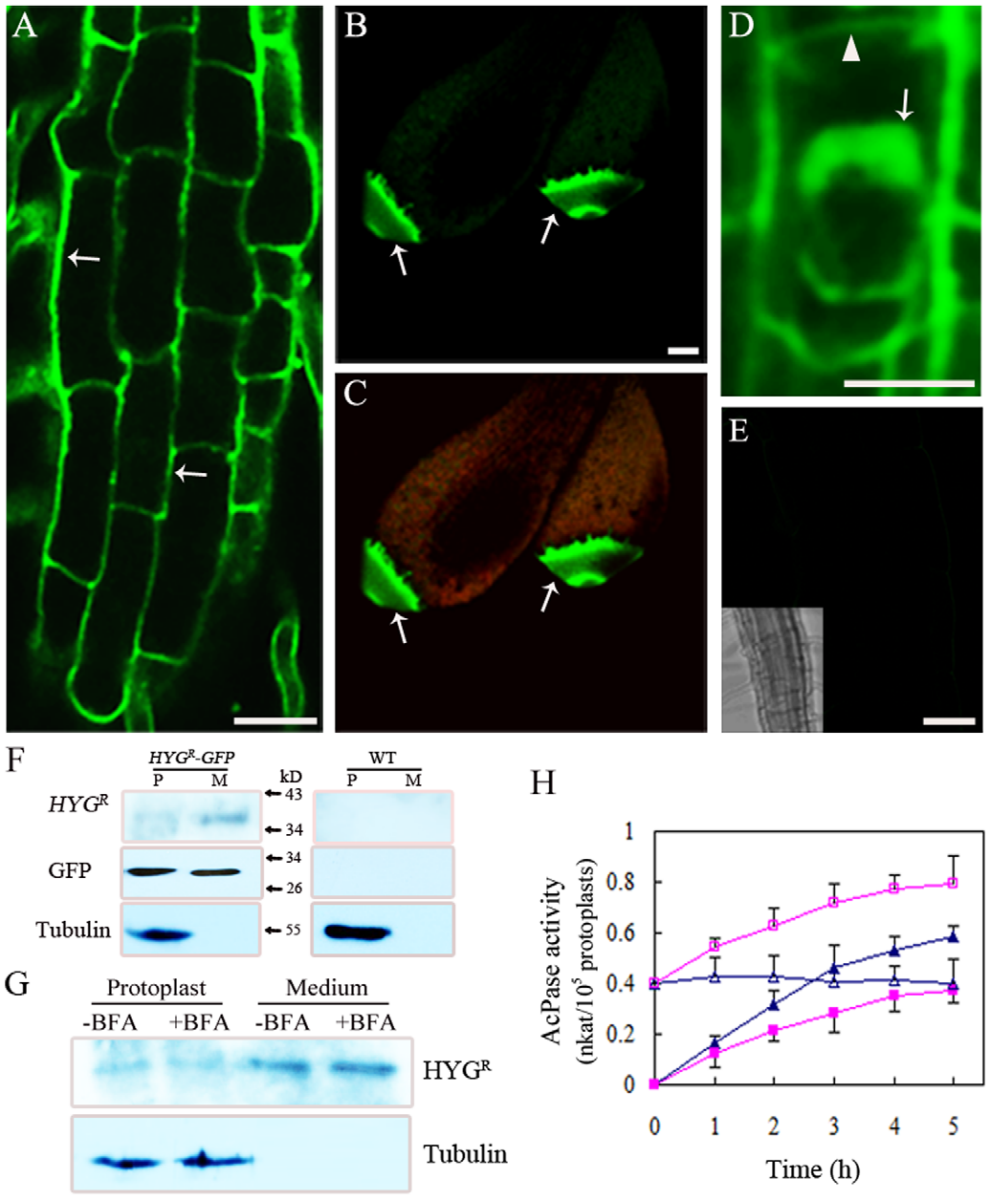
Subcellular localization of HYG^R^-GFP and secretion of HYG^R^ in transgenic *Arabidopsis*. (A) Confocal image of transgenic root cells showed that HYG^R^-GFP was localized on the cell surface (arrows). Bar = 20 µm. (B and C) Confocal image of transgenic leaf showed that HYG^R^-GFP (green) was primarily expressed in the leaf-tip zone cells (arrows). Autofluorescence of chloroplasts appear as red structures. Bar = 100 µm. (D) Confocal image showed that HYG^R^-GFP was localized in both cell wall (arrows) and cytoplasm (arrowheads). The root cells were plasmolysed with 0.8 M mannitol for 1 h. Bar = 20 µm. (E) Control image of non-transgenic root cells taken at the same laser intensity and exposure time as that in (A) and (D). Inset is the reduced bright field image. Bar = 30 µm. (F) Non-transgenic protoplasts (WT) and protoplasts stably expressing *HYG^R^-GFP* were incubated for 5 h at 23°C. The protoplasts lysates (P) and medium (M) proteins were subjected to protein gel blot with anti-HYGR, anti-GFP and anti-tubulin antibody, respectively. (G) The response of HYG^R^ secretion to BFA treatment. Protoplasts stably expressing *HYG^R^-GFP* were incubated for 5 h with (+) BFA or without (−) BFA at 23°C. The protoplasts lysates and medium proteins were separated by SDS-PAGE and immunoblotted with anti-HYG^R^and anti-tubulin antibody, respectively. (H) The effectiveness of bredeldin A (BFA) was demonstrated by the variation of acid phosphatase (AcPase) activities. Protoplasts were incubated in the absence (▵, ▴) or presence (□, ▪) of BFA. At the indicated periods during incubation, the protoplast was separated from the medium by centrifugation. AcPase activities in the medium (▴, ▪) and protoplasts fractions (▵, □) were determined. Note that the partial inhibition of the activities of AcPase after BFA treatment.

To obtain the clues as to why co-expression of SYT2-GFP and HYG^R^ caused hypersensitivity to hygromycin B in *Arabidopsis*, we first analyzed the subcellular localization of a translational fusion between HYG^R^ and GFP under the control of the constitutive promoter (*CaMV35S*) in stable transgenic lines. The fluorescent signals from HYG^R^-GFP were present on the cell surface of root cells and interestingly HYG^R^-GFP was preferentially expressed in leaf-tip zones (Figure 6A to 6C). In the plasmolyzed cells, HYG^R^-GFP was found in the cytoplasm as well as in the cell walls (Figure 6D and 6E). As it has been mentioned that the HYG^R^ protein could be secreted in plants [35], we first investigated the secretory property of the HYG^R^-GFP protein by protein gel blot using mesophyll protoplasts of *HYG^R^-GFP* plants. Unexpectedly, in protoplast lysates, the expected band of full length HYG^R^-GFP (about 65 kD) was not detected when anti-HYG^R^ antibody was applied, but a band with a molecular weight between 34–43 kD (about the molecular weight of HYG^R^ protein) was detected both in the medium and in the protoplast lysates (Figure 6F), suggesting that HYG^R^ protein was secreted into extracellular space. When anti-GFP antibody was applied, the HYG^R-^GFP protein appeared as a single band of approximately 30 kD, slightly bigger than GFP, in both the medium and the protoplast lysates of *HYG^R-^GFP*-expressing plants. Using tubulin as an intracellular marker, it was found that contamination of the medium with intracellular proteins was below the level of detection. These results suggest that HYG^R^-GFP had been efficiently truncated at carboxyl terminus of HYG^R^ shortly after it was synthesized in *HYG^R-^GFP*-expressing plants. To assess whether HYG^R^-GFP was secreted via the conventional secretory pathway, protoplasts were treated with BFA. Although the Golgi apparatus in *Arabidopsis* root tissues appears to be BFA-resistant [32]–[34], it turns out that BFA indeed exerts a marked effect on the Golgi apparatus in non-root tissues of *Arabidopsis*. Confocal microscopy revealed that the classic re-absorption of Golgi membranes back into the ER in BFA-treated *Arabidopsis* leaves [34], [36]. Furthermore, the secretion of acid phosphatase inhibited by BFA treatment was also reported in mesophyll protoplasts of tobacco, indicating that BFA inhibits the conventional secretory pathway in leaf cells [37]. In order to analyze whether BFA affected the secretion of HYG^R^-GFP by immunoblot analysis, the total protein of protoplast lysates and the medium was harvested after 5-h BFA treatment, respectively. As shown in Figure 6G, HYG^R^ was detected in similar amounts in the absence and presence of BFA in the protoplast lysates or in the medium, respectively. The effectiveness of BFA on the conventional ER/Golgi pathway was verified by measuring the activity of acid phosphatase (AcPase) at hourly intervals in the medium and protoplast lysates (Figure 6H). As expected, an obvious inhibition of AcPase secretion upon BFA treatment was observed at each individual measurement time as previously reported [37]. The facts that BFA inhibited AcPase secretion but did not inhibit secretion of HYG^R^-GFP suggested that HYG^R^-GFP secretion indeed follows an alternative secretory pathway.

### SYT2 is Required for the Unconventional Secretion of HYG^R^

**Figure 7:**
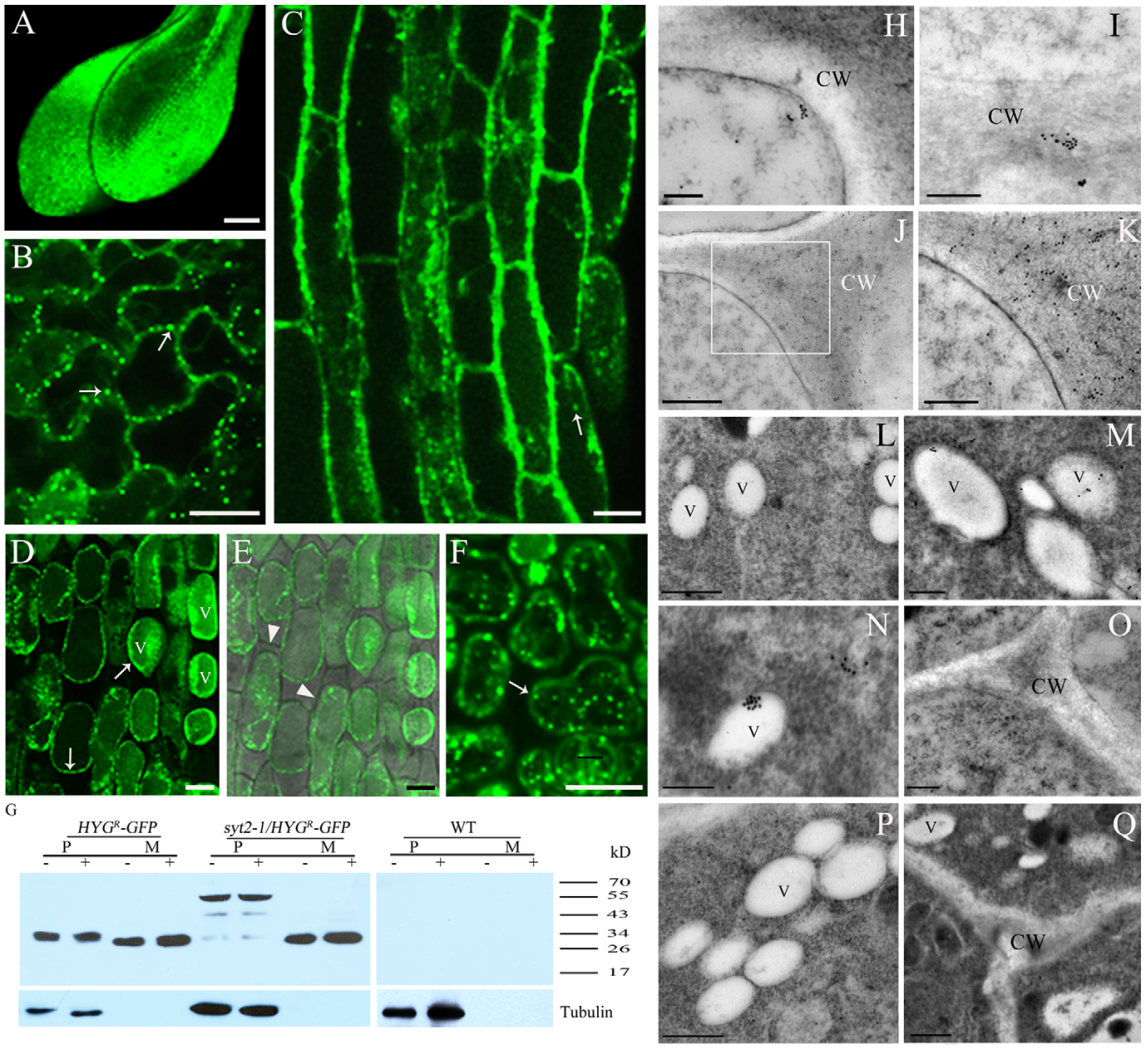
Accumulation of HYG^R^ in *syt2-1* plants. (A–C) Expression of HYG^R^-GFP in *syt2-1* caused the fluorescent accumulation in cytoplasm. In magnified pictures punctate structures were found (B and C; Bars = 100 µm). Bar = 20 µm (A). (D–F) Confocal image showed that HYG^R^-GFP was brightly accumulated in punctate structures in cytoplasm (arrowheads) and in vacuoles. The cells were plasmolysed with 0.8 M mannitol for 1 h. V: vacuole. Bars = 20 µm. (G) Protein gel blot of HYG^R^-GFP in wild-type (WT), *syt2-1*, and *HYG^R^-GFP* plants. The blots were probed with anti-GFP (upper) and anti-tubulin (lower) antibodies to detect GFP-tagged proteins and tubulin, respectively. The positions of molecular weight markers are indicated. (H–K) Immuno-gold labeling and electron microscopic observation showed that HYG^R^ was detected on the cell wall in root tip cells of HYG^R^-GFP-expressing *Arabidopsis* plants. (H and I) Concentrated gold particles near/on the cell wall. (J and K) The distribution of gold particles on the cell wall. (K) The magnification image of the inset in (J). CW: Cell wall. Bars: 500 nm (H, J), 100 nm (I), 200 nm (K). (L–O) Immuno-gold labeling of HYG^R^ in the root tip cells of *syt2-1* plants expressing *HYG^R^-GFP*. (L–N) HYG^R^ was detected in/on vacuoles. (O) No signals were found in the cell wall. CW: Cell wall. V: Vacuole. Bars: 500 nm (L), 200 nm (M, O), 100 nm (N). (P and Q) Immuno-gold labeling using anti-HYG^R^ antibody in the root tip cells of *HYG^R^-GFP*-expressing plants (P) and non-transgenic plants (Q). CW: Cell wall. V: Vacuole. Bars: 200 nm (P), 100 nm (Q).

To further examine whether SYT2 was involved in the unconventional secretory process of HYG^R^-GFP in *Arabidopsis*, the HYG^R^-GFP was introduced into *syt2-1* plants by *Agrobacterium*-mediated transformation and the resultant transgenic plants (*syt2-1/HYG^R^-GFP*) had similar phenotype to *syt2-1/HYG^R^* plants under hygromycin B treatments (Figure S6). As shown in Figure 7A to 7C, expression of HYG^R^-GFP resulted in an increase in GFP fluorescence owing to intracellular accumulation of HYG^R^-GFP. HYG^R^-GFP accumulated in whole leaf cells besides in leaf-tip zones (Figure 7A). When we examined the tissues at higher resolution by LSCM, the fluorescence signals were found on punctate structures in cytoplasm (Figure 7B and 7C). After being plasmolyzed, *syt2-1/HYG^R^-GFP* plants showed that fluorescence signals of *HYG^R^-GFP* were primarily localized on intracellular punctate structures and vacuoles (Figure 7D to 7F). To characterize the fluorescent proteins in *syt2-1/HYG^R^-GFP* plants, total proteins extracted from protoplasts and medium were analyzed by protein gel blot using anti-GFP antibody. As shown in Figure 7G, the total protein in the medium contained no detectable tubulin, indicating that the medium was not obviously contaminated by protoplast proteins. HYG^R-^GFP in the medium extracts of *syt2-1/HYG^R^-GFP* protoplasts similarly exhibited a single band that co-migrated with the GFP-fusion protein in the extracts from *HYG^R^-GFP* protoplasts and medium. However, the protoplast extracts had three forms of GFP fusion protein in *syt2-1/HYG^R^-GFP* plants. The greatest band migrated with an apparent molecular weight of about 55 kD which was lower than the expected full-length of HYG^R-^GFP. Apart from this upper GFP fusion protein, two less intense bands with the molecular weight of about 43 kD and 30 kD were recognized below it, implying that HYG^R^-GFP had undergone partial truncation at its amino terminus with different extents in the *syt2-1/HYG^R^-GFP* plants. Immunogold-labeled ultrathin sections for electron microscopy showed the gold particles situated on the cell wall both in concentrated and dispersed manner in the root cells of *HYG^R-^GFP*-expressing plants (Figure 7H to 7K). Little or no gold particles were detected on the cell wall in the root cells of the *syt2-1* plants expressing *HYG^R-^GFP*. However, several gold particles well deposited close to, or in the vacuoles in these cells (Figure 7L to 7O). No obvious signals were found in the vacuoles of the *HYG^R-^GFP* transgenic plants (Figure 7P) or in the whole cells of non-transformed plants (Figure 7Q).

It was of interest to note that *syt2-1/HYG^R^* seedlings also exhibited the inhibition of root elongation under higher hygromycin B treatments (>5 µg/ml) (Figure 5). To confirm that the sensitivity of *syt2-1/HYG^R^* to hygromycin B in root tip growth is caused by the deficiency of SYT2, the binary construct containing *SYT2-GFP* and *HYG^R^* was introduced into *syt2-1* mutants by *Agrobacterium*-mediated transformation. T3 progeny were subjected to hygromycin B and it was found that the *SYT2/HYG^R^* plants restored the *syt2-1* phenotype to the *HYG^R^ transgenic* plants with respect to the root elongation and root morphology (Figure S7). These results confirmed that the deficiency of SYT2 in *syt2-1* resulted in the increased sensitivity to higher concentrations of hygromycin B during *Arabidopsis* seedling growth.

### *SYT1* Expression is Up-regulated in Hygromycin B-treated *syt2-1* Mutant

**Figure 8:**
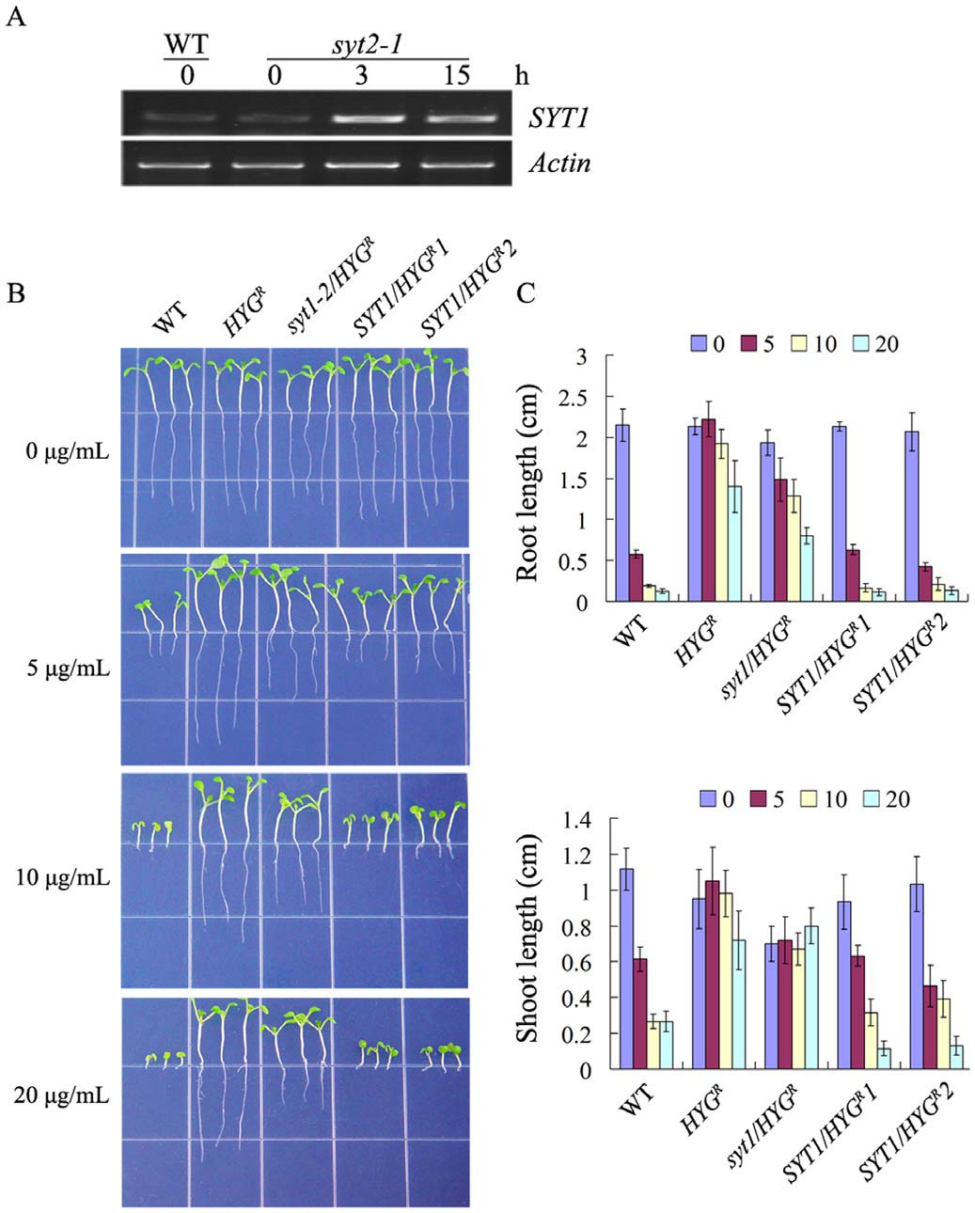
Response of *SYT1*-overexpressing plants to hygromycin B. (A) Reverse transcriptase-PCR analysis of *SYT1* transcripts in wild-type (WT) and *syt2-1* plants after hygromycin B treatments for 0, 3 and 15 h. *Actin* was used as a control. (B) Growth of wild-type (WT), *HYG^R^*, *syt1-2*/*HYG^R^ and SYT1/HYG^R^* plants under hygromycin B treatments. Seeds were germinated on ½ MS medium supplemented with indicated concentrations of hygromycin B and grew for 7 days before images were taken. *SYT1/HYG^R^1* and *SYT1/HYG^R^2* were different lines that were simultaneously transformed with *SYT1* and *HYG^R^* genes. (C) Measurement of length of roots and shoots for seedlings treated as described for (B). Values are the means ± SD of 30–40 seedlings from three independent experiments.

The detoxifying ability of HYG^R^ in the *syt2-1* mutant was reduced under higher concentrations of hygromycin B when compared with that in the wild-type plants, but was much higher than that in SYT2-overexpressing plants. We hypothesized that the other members of SYT family in *Arabidopsis*, especially the SYT1, which has the highest homology with SYT2, might contribute to the decreased resistance of *syt2-1* to hygromycin B. To address this possibility, the expression level of *SYT1* in hygromycin-treated *syt2-1* plants was examined by semi-quantitative RT-PCR. Under normal condition, *SYT1* is expressed at similar level in *syt2-1* to that in wild-type plants. However, the expression of *SYT1* was greatly enhanced in *syt2-1* plants under hygromycin B treatment for 3 h and 15 h (Figure 8A). To further examine whether the up-regulated expression level of *SYT1* in *syt2-1* has a role of enhancing the sensitivity to hygromycin B, we investigated the phenotype of *syt1-2* (*SYT1* knock-out mutant, Schapire et al., 2008) and *SYT1*-overexpresing plants both which contain *HYG^R^* gene. From Figure 8B and 8C, it is evident that co-expression of *SYT1* and *HYG^R^* led to hypersensitivity to hygromycin B in *Arabidopsis*.

## Discussion

Like most proteins involved in vesicular trafficking, the localization of synaptotagmins provides important information about the biological functions of these proteins [38]. Thus, we firstly investigated the subcellular distribution of SYT2 using different approaches. In all cases, SYT2 was detected in punctate structures, raising the possibility that it was targeted to the membrane trafficking pathway. This was further supported by the results obtained from immunolocalization studies using anti-SYT2 and anti-GFP antibodies. These studies showed that SYT2 was broadly distributed on the Golgi apparatus. This result is analogous to mammalian Syt 4, which localizes to the Golgi apparatus in undifferentiated neuroendocrine PC12 cells [39]. However, the localization of SYT2 is in contrast to the plasma membrane localization of SYT1 in *Arabidopsis* [14].

It has been long appreciated that the Golgi apparatus forms the heart of the secretory pathway and it is where secretory materials are posttranslationally modified before being sorted for delivery to their final destination, such as the plasma membrane or extracellular space [40], [41]. Indeed, in plant cells, some Golgi-localized proteins have been shown to be involved in the transport of cargo from the Golgi apparatus to the cell surface [42], [43]. Therefore, the localization of SYT2 on the Golgi apparatus in *Arabidopsis* suggests a role in the secretory pathway.

**Figure 9:**
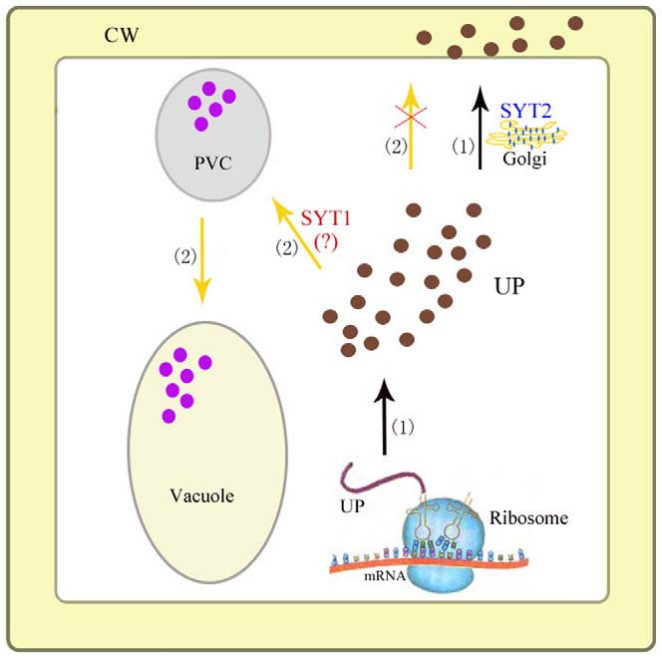
Hypothetical model summarizing function of SYT2 in trafficking of the unconventional proteins (UPs) in *Arabidopsis*. (1) UPs were synthesized on the free ribosomes in the cytoplasm and then secreted into the cell wall (CW). Golgi apparatus-localized SYT2 is involved in their secretion. (2) When *SYT2* gene gets knocked out, a proportion of the UPs trafficks through prevavuole compartment (PVC) en route to the vacuoles. SYT1 is probably involved in the redirectional trafficking of UPs in the *syt2-1* mutant. (?) indicates the speculated role of SYT1 in the *syt2-1* plants.

HYG^R^ has been shown to be effective in selection with various plant species, including dicots, monocots and gymnosperms [44], [45]. However, the data available at present appear insufficient to provide complete knowledge of mechanism of HYG^R^ secretion. In the present experiment, HYG^R^-GFP was present in both intracellular and extracelluar space, suggesting that HYG^R^-GFP may be excreted from cytosol into the extracellular space. Furthermore, anti-GFP antibodies recognized a band with a molecular weight of about 30 kD in the protoplast lysates from *HYG^R^-GFP* plants, which was slightly greater than the full-length GFP, implying that *HYG^R^-GFP* was truncated at the carboxyl terminus of HYG^R^ shortly after its synthesis and HYG^R^-GFP was secreted in its truncated form. Interestingly, co-expression of *HYG^R^* and *SYT2* in *Arabidopsis* caused hypersensitivity to hygromycin B, suggesting that SYT2 may have a role in regulating the detoxification of HYG^R^ for hygromycin B. To confirm whether SYT2 is involved in the trafficking of HYG^R^, we examined the existing form of HYG^R^-GFP in *syt2-1* mutant. We found that the loss of SYT2 partially inhibited the truncation of HYG^R^-GFP at the carboxyl terminus of HYG^R^, which subsequently accumulated in intracellular punctate structures and vacuoles in several truncating forms, suggesting SYT2 has a vital role in regulating the trafficking of HYG^R^-GFP for its secretion in plant cells (Figure 9).

Proteins can be secreted in plant cells via either the conventional or the unconventional secretory pathway. The unconventional secretory proteins not only lack of canonical signal sequence, but are also resistant to the export processes affected by BFA, an inhibitor of ER/Golgi-dependent protein secretion in both animals and plants [4], [28], [46]. Interestingly, no conventional signal peptide sequence was found in HYG^R^ predicted by SignalP 3.0 Server (http://www.cbs.dtu.dk/services/SignalP/) or TargetP 1.1 Server (http://www.cbs.dtu.dk/services/TargetP/). Therefore, it is possible that HYG^R^-GFP was synthesized on the free ribosomes in cytoplasm and exported by a signal peptide-independent secretory process (Figure 9). This unconventional secretion has been thoroughly demonstrated in mammalian and yeast cells. We further analyzed the secretion of HYG^R^-GFP protein in *HYG^R^*-*GFP*-expressing plants in response to BFA treatment by protein immunoblot. As expected, upon BFA treatment, HYG^R^-GFP secretion in the transgenic *Arabidopsis* was not perturbed. Thus HYG^R^-GFP secretion displays the features of leaderless or unconventional protein secretion, including the absence of a canonical signal peptide in the protein and the insensitive export in the presence of brefeldin A [4], [46], Therefore, we can safely conclude that an unconventional secretion is involved in *HYG^R^*-transgenic *Arabidopsis* plants.

It is unexpected that the detoxifying ability of HYG^R^ in the loss-of-function *syt2-1* mutant was also destroyed, causing the plants to grow slowly and weakly under higher concentrations of hygromycin B, although these plants showed stronger resistance compared with *SYT2/HYG^R^* ones. The most probable explanation for this phenomenon is that the trafficking of HYG^R^-GFP in *syt2-1* plants is inhibited and the protein is partly transported into vacuoles, as revealed by the localization of HYG^R^-GFP in this mutant, and may then be degradated. Co-expression of *HYG^R^* and *SYT1*, the latter, the most similar member to *SYT2* in *Arabidopsis SYT* family, also led to hypersensitivity to hygromycin B. In any case, this result provided direct evidence that the contributor to the weakened resistance of *syt2-1* to hygromycin B might be SYT1. We further found that the transcriptional expression of *SYT1* in *syt2-1* plants remained unchanged under normal conditions, but obviously enhanced under hygromycin B treatment. The SYT1 knock-out mutants also showed sensitivity to hygromycin B even at lower concentration (5 µg/mL), although they have much stronger tolerance than *SYT1/HYG^R^* plants. Considering the similar responses of SYT2 and SYT1 to hygromycin B, we conclude that SYT1 may contribute to the resistance of *HYG^R^*-harboring plants via a different secretory route (Figure 9).

Unconventional secretion can be classified into non-vesicular and vesicular mechanisms. Non-vesicular mechanisms are based on direct translocation of cytoplasmic proteins across the plasma membrane via a specific plasma membrane ATP-binding cassette transporter or some lipids, such as phosphatidylinositol 4,5 bisphosphate [PI(4,5)P_2_] in the inner leaflet of the plasma membrane [47].Vesicular mechanisms of unconventional secretion involved multi-vesicular bodies and exosomes that need to fuse with plasma membranes to release cargo into the extracellular space [47], [48]. In our study, SYT2 was not presented on the multivesicular bodies (PVC in plant cells), indicating that SYT2 protein may regulate the unconventional secretory pathway by a distinct manner from the multivesicular body-mediated secretion of exosome in mammalian cell. However, SYT2 is not the only Golgi-localized protein that regulates unconventional secretion. Golgi-localized protein GRASP (Golgi reassembly stacking protein) in *Dictyostelium discoideum*, is also required for Golgi-independent cell-surface transport of a non-signal-peptide-containing protein, acyl-CoA binding protein (AcbA), which triggers terminal differentiation of spore cells [49], [50]. In *Drosophila melanogaster*, GRASP modulates Golgi-independent cell surface transport of α intergrin. In a *D. melanogaster grasp* mutant, the α integrin subunits are not properly deposited at the plasma membrane and instead retained intracellularly [51]. From sequence comparison of all the available genomes, it was revealed that plants lack a bona fide GRASP homolog [49]. Very recently, an *Arabidopsis* protein Exo70E2 was found to be present in some double membrane structures (named EXPO) and did not colocalize with the Golgi apparatus, the TGN or PVC. Exo70E2 served to release a leaderless protein (SAMS2) into the extracelluar space [52], indicating that there may be diverse proteins which localize on different organelles and modulate the release process of unconventional proteins in plant cells. Therefore, SYT2 is the first protein, to our knowledge, that resides on Golgi apparatus and regulates unconventional protein secretion in plants.

## Methods

### Plant Material and Growth Conditions

Seeds expressing ARA6-GFP and ARA7-GFP were kindly provided by Takashi Ueda and Thierry Gaude [29], [53]. Construct of ST-YFP was kindly made available by Jingbo Jin [54]. *Arabidopsis* mutant *syt2-1* (SALK_135307) was obtained from the *Arabidopsis* Biological Resource Center at Ohio State University. Other transgenic plants were generated based on the protocol in Text S1.

*Arabidopsis* seeds were pretreated in 70% ethanol for 5 min, surface-sterilized in 50% bleach for 1 min, and washed with sterile distillated water at least five times. Seeds were planted on 1% agar containing ½ MS salts with or without the indicated concentrations of hygromycin B, allowed to imbibe for 3 days at 4°C, and germinated in a vertical orientation. Seedlings were grown at 22±3°C under a 16-h light/8-h dark regime. Experiments were performed using 3- to 4-day-old seedlings for microscopic observation, or 7- to 10-day-old seedlings for measurement of root and shoot lengths.

### Antibody Preparation and Protein Gel Blot Analysis

For protein gel blot analysis, a polyclonal antibody was raised against a truncated form of SYT2. The C-terminal region of SYT2 (235 amino acid residues) and the full length of HYG^R^ were expressed in *E. coli* respectively as recombinant proteins using the expression vector pET28b (Invitrogen, Carlsbad, CA). The recombinant proteins were expressed and purified according to the manufacturer's protocol, and the purified proteins were injected into a rabbit to raise antibody according to a published protocol [55]. The polyclonal antibody was purified according to Park et al. [56]. Monoclonal anti-GFP antibodies were purchased from Sigma-Aldrich (St. Louis, MO).

Total protein extracts were obtained by grinding 100 mg of wild-type, *syt2-1*, or SYT2-GFP-overexpressing plants in protein extraction buffer [20 mM Tris-HCl, pH 7.5, 5 mM ethylenediaminetetraacetic acid (EDTA), 5 mM ethylene glycol tetraacetic acid (EGTA), 10 mM dithiothreitol (DTT), 0.05% sodium dodecyl sulfate (SDS), and 1 mM phenylmethylsulfonyl fluoride (PMSF)]. The extracts were spun for 10 min at 4°C, and the resulting supernatant loaded on a SDS-PAGE gel with loading buffer. For HYG^R^ protein hybridization, mesophyll protoplasts were prepared from the leaf tissues of 3- to 4-week-old *Arabidopsis* plants which stably expressed HYG^R^-GFP protein [57]. After being washed five times with W5 solution (154 mM NaCl, 125 mM CaCl_2_, 5 mM KCl, 2 mM 4-Morpholineethanesulfonic acid, pH 5.7), the protoplasts were incubated with 25 µM BFA for 5 hours. At the end of the incubation, the medium and the protoplasts were collected respectively. The protoplast proteins were extracted as described by Wu et al [58]. The medium proteins were precipitated by trichloroacetic acid method and resolved in the SDS-PAGE loading buffer. The samples were boiled for 10 min and loaded on polyacrylamide gel.

After electrophoresis, the separated proteins were transferred to a nitrocellulose membrane for 2 h. The nitrocellulose membrane was then incubated in a 1∶800 dilution of anti-SYT2, 1∶500 anti-HYG^R^, 1∶1000 anti-tubulin or 1∶4000 anti-GFP antibodies in phosphate-buffered saline (PBS) buffer (pH 6.9). Horseradish-peroxidase-conjugated secondary antibody (Sigma-Aldrich) was used at a 1∶5000 dilution, and the results were interpreted using an enhanced chemiluminescence detection system, with visualization by enhanced chemiluminescence detection reagents (Applygen Technologies Inc., Beijing, China) according to the manufacturer's recommendations.

### Fluorescent Dye and Treatments with BFA and Wortmannin

To visualize putative endosomes, seedlings were mounted in ½ MS liquid with 3 µM FM4-64 [Invitrogen; T13320; diluted from a 3 mM stock solution in dimethyl sulfoxide (DMSO)] on slides for a specified time. For BFA treatment, seedlings were incubated in ½ MS liquid containing 25 µM BFA diluted from a 50 mM stock solution in DMSO and then mounted on slides in the presence of BFA. For the wortmannin treatment, seedlings were incubated in ½ MS liquid containing 20 µM wortmannin diluted from a 20 mM stock solution in DMSO for 1 h before observation.

### Immunofluorescent Labeling

Four-day-old seedlings were fixed in 4% paraformaldehyde in PEM buffer (50 mM PIPES, 5 mM EGTA, and 5 mM MgSO_4_, pH 6.9) for 1 h at room temperature, followed by washing with 0.1 M glycine in PEM buffer. Fixed cells were partially digested with 2% (w/v) driselase (Sigma-Aldrich) for 30 min at 37°C. The plasma membrane was permeabilized with 0.3% Triton X-100 and 10% DMSO in PBS for 1 h at room temperature. Seedlings were incubated in blocking solution for 1 h at room temperature and then incubated with primary antibodies of anti-SYT2 (1∶50) or anti-GFP (1∶200; Sigma-Aldrich) again for 1 h at room temperature. Primary antibodies were washed out with blocking solution three times for 5 min and the seedlings then incubated with fluorochrome-conjugated secondary antibodies in the dark at 37°C for 3 h. Secondary antibodies (purchased from Sigma-Aldrich) were used at the following concentrations: fluorescein isothiocyanate-conjugated anti-rat IgG, 1∶100; fluorescein isothiocyanate-conjugated anti-rabbit IgG, 1∶100; rhodamine (TRITC)-conjugated anti-rabbit IgG, 1∶100.

### Fluorescence Microscopy

Fluorescence microscopy was performed using a TCS SP5 confocal laser-scanning microscope (Leica, Oberkochen, Germany). All LSCM images were obtained using the Leica Confocal software and a 63× water-immersion objective. GFP or GFP/FM4-64 was excited at 488 nm and emission was detected between 500 and 530 nm for GFP and between 620 and 680 nm for FM4-64. To visualize GFP/RFP, GFP and RFP were excited at 488 nm and 543 nm, respectively, and emission detected at 500 and 530 nm for GFP and between 565 and 600 nm for RFP. Images were edited using the LAS AF Lite image browser (Leica) and Adobe Photoshop CS3 (Adobe Systems, San Jose, CA).

### Immunoelectron Microscopy

For immunogold labeling of SYT2 and HYG^R^, roots of *Arabidopsis* were fixed with 4% paraformaldehyde and 1% glutaraldehyde for 4 h and then were embedded in LR White resin (Sigma) and polymerized by heat. Ultrathin sections were obtained and transferred to nickel grids that were then blocked with 5% BSA and incubated subsequently with the primary anti-body (anti-SYT2, 1∶200; anti-HYG^R^, 1∶200) at 37°C for 1 h. After five washes with PBS for 20 min, the sections were treated with the secondary antibody (goat anti-rabbit IgG coupled to 10-nm gold particles, Sigma, 1∶50) at 37°C for 1 h. Finally, the sections were stained with 2% uranyl acetate for 10 min and observed under JEM-1230 TEM (JEOL).

### Acid Phosphatase Assay

The activity of the AcPase was measured according to Pfeiffer by measuring the release of *p*-nitrophenol (*p*NP) from *p*-nitrophenyl phosphate (*p*NPP) [59]. Samples of 200 µl were incubated with 200 µl of reaction buffer containing 40 mM MES-Tris, pH 5.5, 5 mM *p*NPP, and 10 mM MgCl_2_, for 45 min at 30°C. The reaction was stopped by the addition of 5 ml of 40 mM NaOH, and the concentration of *p*NP was determined at 405 nm wavelength. All assays were performed as triplicate.

## Supporting Information

Text S1 **Material and Methods (Construction of Chimeric Genes and** Transformation of *Arabidopsis* Plants, **RT-PCR Analysis, Transient Expression in Tobacco and** ***Arabidopsis*** **).**
(DOCX)

Figure S1 **Expression profiles of** ***Arabidopsis SYT2*** **based on microarray expression data from Genevestigator (** https://www.genevestigator.com **).**
(TIF)

Figure S2 **No co-localization between SYT2-GFP and BFA compartments.** (A–F) Seedlings stably expressing SYT2-GFP were stained with FM4-64 for 10 min and treated with either DMSO (A–C) or 25 µM BFA for 60 min (D–F). FM4-64-labeled BFA compartments (red) mostly non-overlapped with SYT2-GFP punctuate structures (green). Arrows indicate BFA compartments. Bars = 10 µm. (G–L) Seedlings stably expressing VHA-a1-GFP or ARA6-GFP were stained with FM4-64 for 10 min before being treated with 25 µM BFA for 60 min. VHA-a1-GFP-labeled structures (green) aggregated and perfectly overlapped with BFA compartments (red) (G–I), and ARA6-GFP-positive structures (green) clustered at the periphery of BFA compartments (red) (J–L). Arrows indicate BFA compartments. Bars = 10 µm.
(TIF)

Figure S3 **SYT2-GFP structures are insensitive to wortmannin.** (A–C) Seedlings stably expressing SYT2-GFP were incubated in FM4-64 (red) for 10 min followed by treatment with 20 µM wortmannin for 60 min. SYT2-GFP-containing structures (arrows) are insensitive to wortmannin. Bar = 10 µm. (D–I) Seedlings containing ARA6-GFP or ARA7-GFP were incubated in FM4-64 (red) for 10 min followed by treatment with 20 µM wortmannin for 60 min. Wortmannin induced the ring-shaped structures (arrows) of ARA6-GFP (D–F) and ARA7-GFP (G–I). Bars = 10 µm. (J–L) Double-labeling with anti-SYT2 and anti-GFP antibodies in root cells containing PVC marker ARA7-GFP. Anti-SYT2 and anti-GFP antibodies were labeled with tetramethylrhodamine-5-isothiocyanate (TRITC)-labeled anti-rabbit IgG and FITC-labeled anti-rat IgG, respectively. Bars = 10 µm.
(TIF)

Figure S4 **Responses of different plants to hygromycin B treatments.** (A–C) Phenotype of plants co-expressing *SYT2-GFP* and *HYG^R^*. Plants were screened on ½ MS medium with 20 µg/mL hygromycin B and then grew for 30 days in soil. Arrows indicate that development of axillary buds due to lacking of apical dominance. (D–H) Phenotypes of *HYG^R^* (D and E), *syt2-1*/*HYG^R^* (F), *SYT2-GFP/HYG^R^* (G) and wild-type (H) plants on hygromycin B-containing medium. Seedlings grew on ½ MS medium supplemented with 0 (D) or 5 µg/mL (E–H) hygromycin B for 3 days before images were taken. Bars = 500 µm. (I–L) Sensitivity of root tips of *HYG^R^* (I), *syt2-1/HYG^R^* (J), *SYT2/HYG^R^* (K) and *syt2-1*/*SYT2/HYG^R^* (L) to hygromycin B. Seeds were germinated and grew on ½ MS medium with 10 µg/mL hygromycin B for 3 days before images were taken. Bars = 100 µm.
(TIF)

Figure S5 **Responses of** ***syt2-1*** **and wild-type seedlings to hygromycin B treatments.** Seeds were germinated on ½ MS medium containing 0, 5 and 10 µg/mL hygromycin B and grown for 7 days before the pictures were taken.
(TIF)

Figure S6 **Responses of** ***HYG^R^-GFP*** **and** ***syt2-1/HYG^R^-GFP*** **seedlings to hygromycin B treatments.** Seeds were germinated on ½ MS medium containing 0, 10 and 20 µg/mL hygromycin B and grown for 10 days before the pictures were taken. *syt2-1/HYG^R^-GFP1* and *syt2-1/HYG^R^-GFP2* were different lines that *HYG^R^-GFP* is expressed in SYT2 knock-out plants.
(TIF)

Figure S7 **Expression of SYT2 complements the** ***syt2-1*** **phenotype under hygromycin B treatment.** (A) Seeds were germinated on ½ MS medium containing 0, 30 and 60 µg/mL hygromycin B and grown for 10 days before the pictures were taken. (B) Measurement of length of roots and shoots for seedlings treated as described for (A). Values are the means ± SD of 30–40 seedlings from three independent experiments.
(TIF)
