# Supplementary material for: Golgi Apparatus-Localized Synaptotagmin 2 Is Required for Unconventional Secretion in Arabidopsis
Source: PLoS One. 2011 Nov 28;6(11):e26477. doi: 10.1371/journal.pone.0026477 (PMC3225361; doi:10.1371/journal.pone.0026477)
Supplement: Text S1 — Material and Methods (Construction of Chimeric Genes and Transformation of Arabidopsis Plants, RT-PCR Analysis, Transient Expression in Tobacco and Arabidopsis ). (DOCX) [file pone.0026477.s001.docx]

**Material and Methods**

**Construction of Chimeric Genes and Transformation of *Arabidopsis* Plants**

The entire coding regions of SYT1 and SYT2 genes were extracted from *Arabidopsis* cDNA by polymerase chain reaction (PCR) using PrimerSTAR HS DNA polymerase (TaKaRa, Otsu, Japan) and the primer pairs SAS (5’-GCGactagtATGGGCTTTTTCAGTACGATA-3’) and SAA (5’-ATAggtaccAGAGGCAGTTCGCCACTCG-3’), SBS (5’-GCGactagtATGGGAATAATCAGCACAAT-3’) and SBA (5’-GAA ggtaccAGAAGAATTTCTCCACTGAAG-3’). The *HYG^R^* coding region was amplified from plasmid pCambia1301 containing *HYG^R^* using the primers HYGS (5’-GACtctagaATGAAAAAGCCTGAACTCACC-3’) and HYGA (5’-ATTggtaccTTTCTTTGCCCTCGGACGAG-3’). The PCR product was digested and subcloned as an *Xba*I/*Kpn*I fragment into *Xba*I/*Kpn*I-digested pCambia1301-*AsMT2b-GFP* (Zhang *et al*., 2006), replacing *AsMT2b* and resulting in pCambia1301-*SYT2-GFP* or pCambia1301-*HYG^R^-GFP*. Sequences of the coding regions were checked by sequencing.

The resulting plasmids were transformed into DH5α competent *E. coli*, and the resulting clones transformed into *Agrobacterium tumefaciens* strain GV3101 by electroporation. Binary vectors were introduced into *Arabidopsis* using the floral dip method (Clough & Bent, 1998). Hygromycin-resistant plants were selected and used for propagating.

**RT-PCR Analysis**

Total RNA was extracted from plant tissue using the RNAsimple Total RNA Kit (TIANGEN BioTECH Inc., Beijing, China). cDNA was synthesized from 2 mg of total RNA treated with DNase I (Promega, Madison, WI) before reverse transcription with AMV reverse transcriptase (TaKaRa). For RT-PCR analysis, 5 μl of single-stranded cDNA from each sample was added to a reaction mixture containing PCR buffers, deoxynucleoside triphosphates, and enzymes. Each sample was then split into three equal aliquots. The sequence-specific primers for *SYT1* (5’- ATGGGCTTTTTCAGTACGATA-3’ and 5’-ATAAGAGACACATAGATATTGGC-3’), *SYT2* (5’- ATGGGAATAATCAGCACAAT-3’ and 5’-GATGGAACCAAAGGCTTCAGAGT-3’), *HYG^R^* (5’-ATGAAAAAGCCTGAACTCACC-3' and 5’- CTATTTCTTTGCCCTCGGACGAG-3’) or actin (5’-TGGTGTCATGGTTGGGATG-3’ and 5’-CACCACTGAGCACAATGTTAC-3’) were designed according to the published cDNA sequences. PCR amplification products were separated in 1% agarose gels and visualized with ethidium bromide.

**Transient Expression in Tobacco and *Arabidopsis***

For *A. tumefaciens*-mediated transient transformation, strain GV3101 (OD_600_=0.5) harboring SYT2-GFP constructs was used for infiltration of *Nicotiana tabacum* L. cv. SR1. and *Arabidopsis thaliana* (Col-0).
