# Supplementary figures and images for: Golgi Apparatus-Localized Synaptotagmin 2 Is Required for Unconventional Secretion in Arabidopsis
Source: PLoS One. 2011 Nov 28;6(11):e26477. doi: 10.1371/journal.pone.0026477 (PMC3225361; doi:10.1371/journal.pone.0026477)

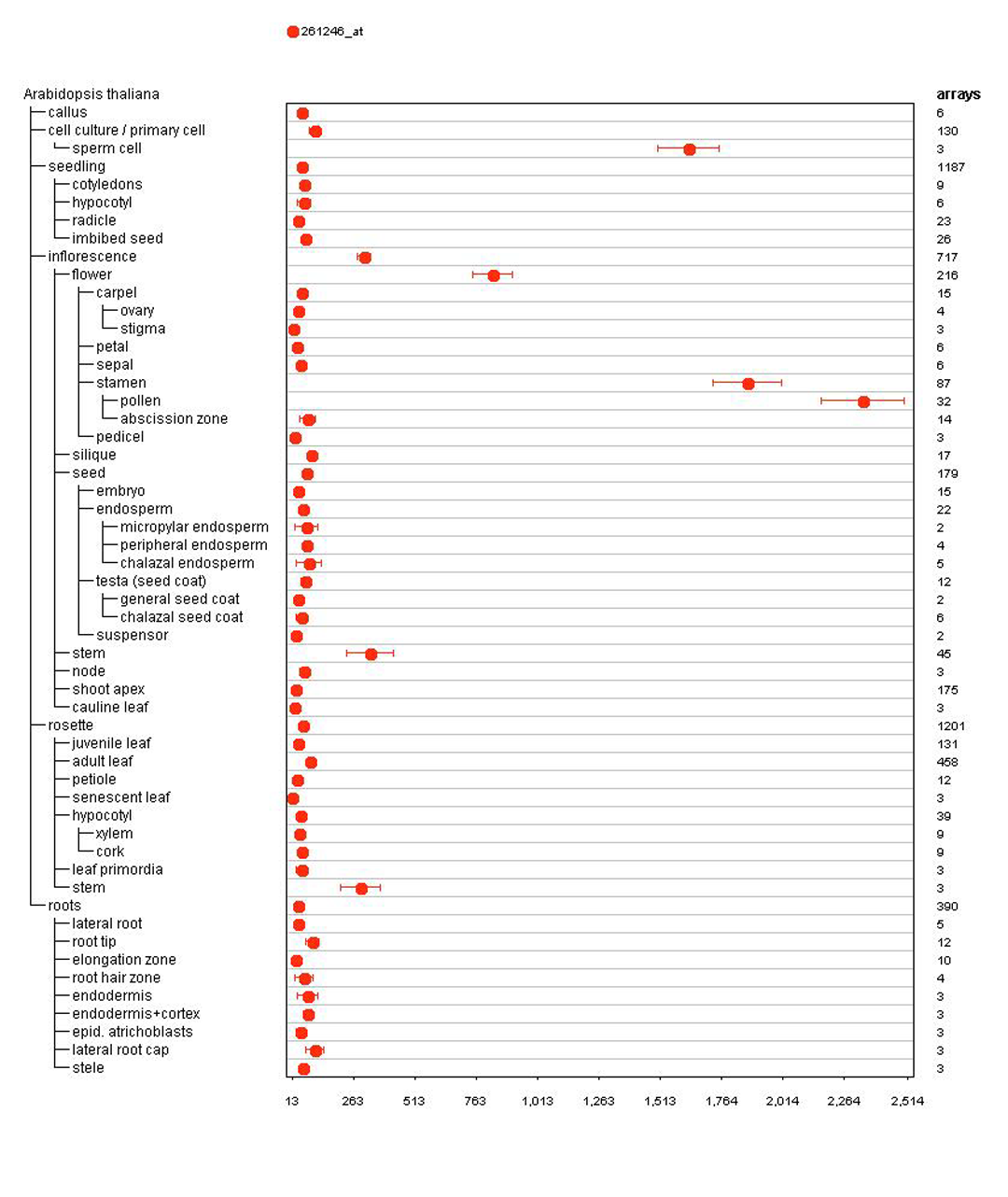

Supplement: Figure S1 — Expression profiles of Arabidopsis SYT2 based on microarray expression data from Genevestigator ( https://www.genevestigator.com ). (TIF) [file pone.0026477.s002.tif]

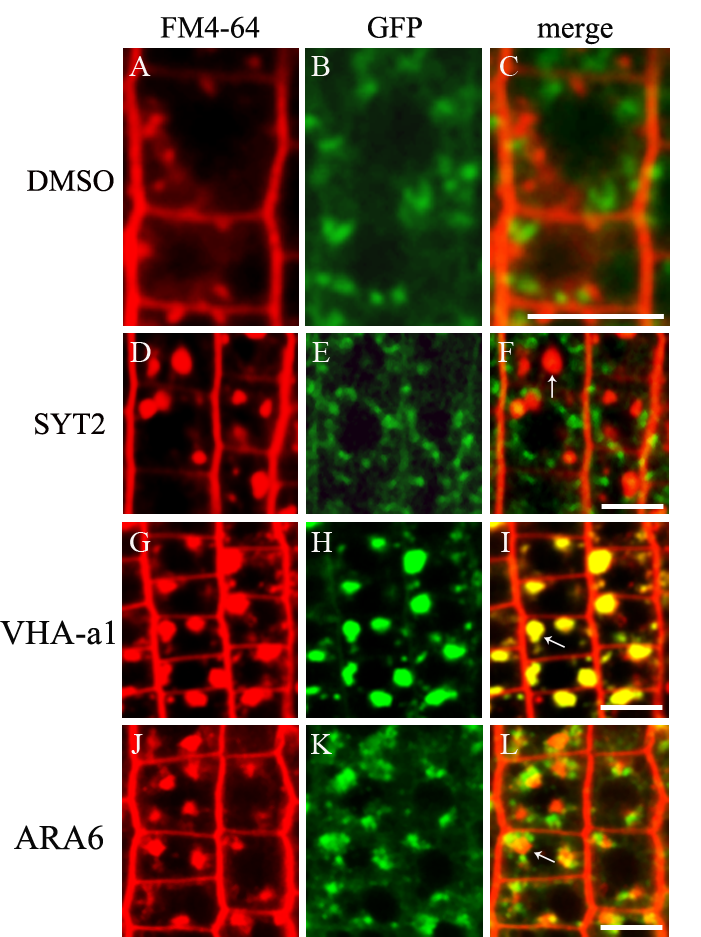

Supplement: Figure S2 — No co-localization between SYT2-GFP and BFA compartments. (A–F) Seedlings stably expressing SYT2-GFP were stained with FM4-64 for 10 min and treated with either DMSO (A–C) or 25 µM BFA for 60 min (D–F). FM4-64-labeled BFA compartments (red) mostly non-overlapped with SYT2-GFP punctuate structures (green). Arrows indicate BFA compartments. Bars = 10 µm. (G–L) Seedlings stably expressing VHA-a1-GFP or ARA6-GFP were stained with FM4-64 for 10 min before being treated with 25 µM BFA for 60 min. VHA-a1-GFP-labeled structures (green) aggregated and perfectly overlapped with BFA compartments (red) (G–I), and ARA6-GFP-positive structures (green) clustered at the periphery of BFA compartments (red) (J–L). Arrows indicate BFA compartments. Bars = 10 µm. (TIF) [file pone.0026477.s003.tif]

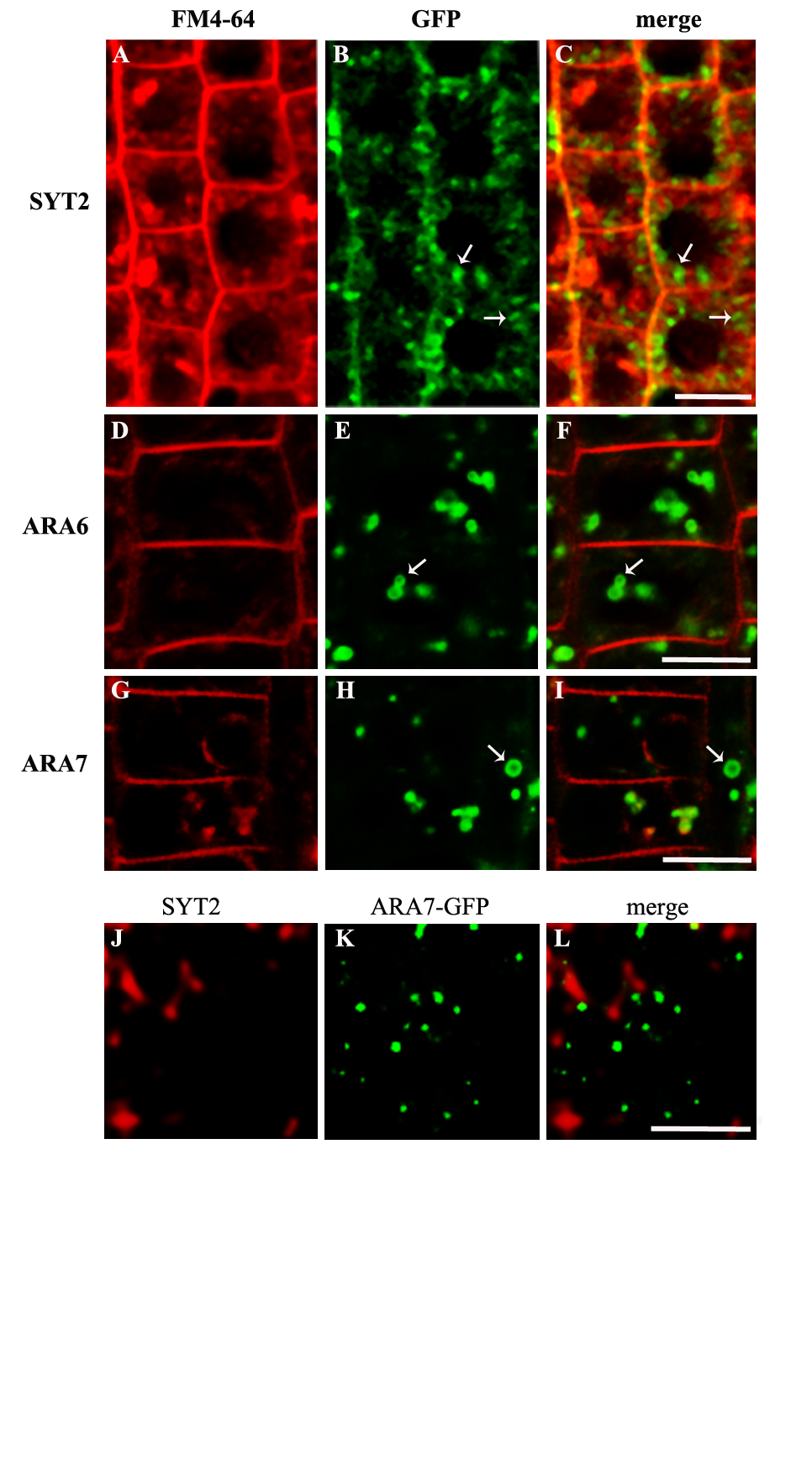

Supplement: Figure S3 — SYT2-GFP structures are insensitive to wortmannin. (A–C) Seedlings stably expressing SYT2-GFP were incubated in FM4-64 (red) for 10 min followed by treatment with 20 µM wortmannin for 60 min. SYT2-GFP-containing structures (arrows) are insensitive to wortmannin. Bar = 10 µm. (D–I) Seedlings containing ARA6-GFP or ARA7-GFP were incubated in FM4-64 (red) for 10 min followed by treatment with 20 µM wortmannin for 60 min. Wortmannin induced the ring-shaped structures (arrows) of ARA6-GFP (D–F) and ARA7-GFP (G–I). Bars = 10 µm. (J–L) Double-labeling with anti-SYT2 and anti-GFP antibodies in root cells containing PVC marker ARA7-GFP. Anti-SYT2 and anti-GFP antibodies were labeled with tetramethylrhodamine-5-isothiocyanate (TRITC)-labeled anti-rabbit IgG and FITC-labeled anti-rat IgG, respectively. Bars = 10 µm. (TIF) [file pone.0026477.s004.tif]

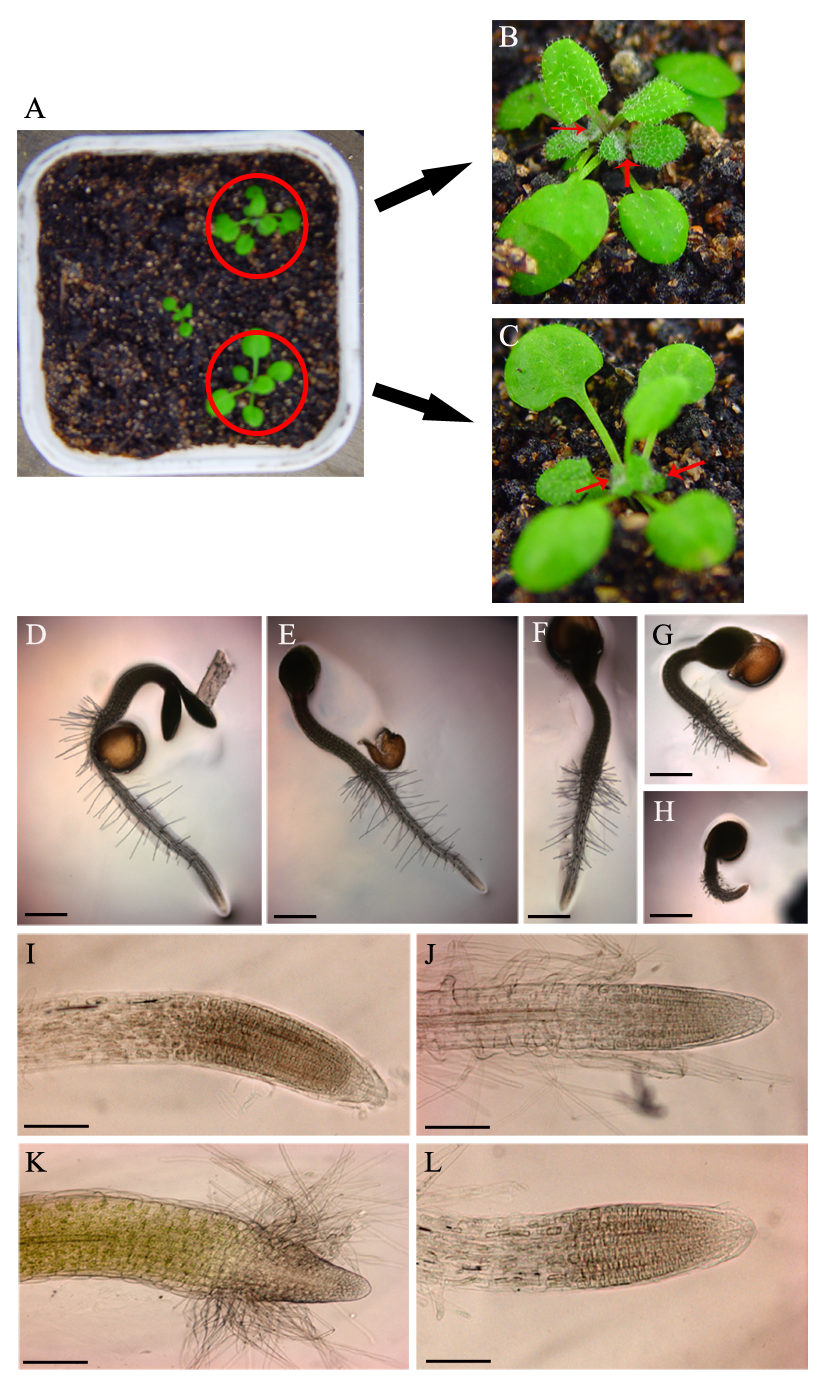

Supplement: Figure S4 — Responses of different plants to hygromycin B treatments. (A–C) Phenotype of plants co-expressing SYT2-GFP and HYGR. Plants were screened on ½ MS medium with 20 µg/mL hygromycin B and then grew for 30 days in soil. Arrows indicate that development of axillary buds due to lacking of apical dominance. (D–H) Phenotypes of HYGR (D and E), syt2-1/HYGR (F), SYT2-GFP/HYGR (G) and wild-type (H) plants on hygromycin B-containing medium. Seedlings grew on ½ MS medium supplemented with 0 (D) or 5 µg/mL (E–H) hygromycin B for 3 days before images were taken. Bars = 500 µm. (I–L) Sensitivity of root tips of HYGR (I), syt2-1/HYGR (J), SYT2/HYGR (K) and syt2-1/SYT2/HYGR (L) to hygromycin B. Seeds were germinated and grew on ½ MS medium with 10 µg/mL hygromycin B for 3 days before images were taken. Bars = 100 µm. (TIF) [file pone.0026477.s005.tif]

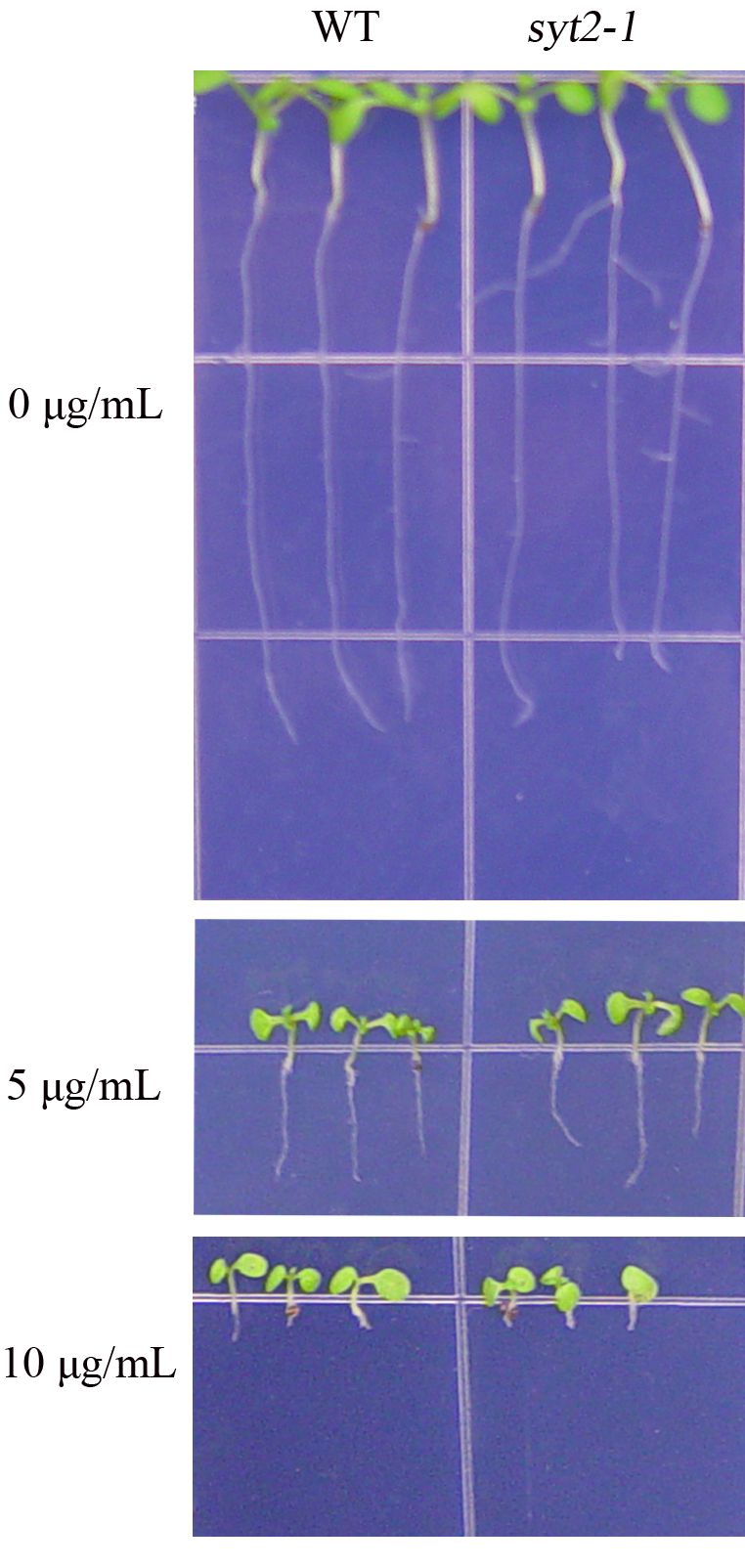

Supplement: Figure S5 — Responses of syt2-1 and wild-type seedlings to hygromycin B treatments. Seeds were germinated on ½ MS medium containing 0, 5 and 10 µg/mL hygromycin B and grown for 7 days before the pictures were taken. (TIF) [file pone.0026477.s006.tif]

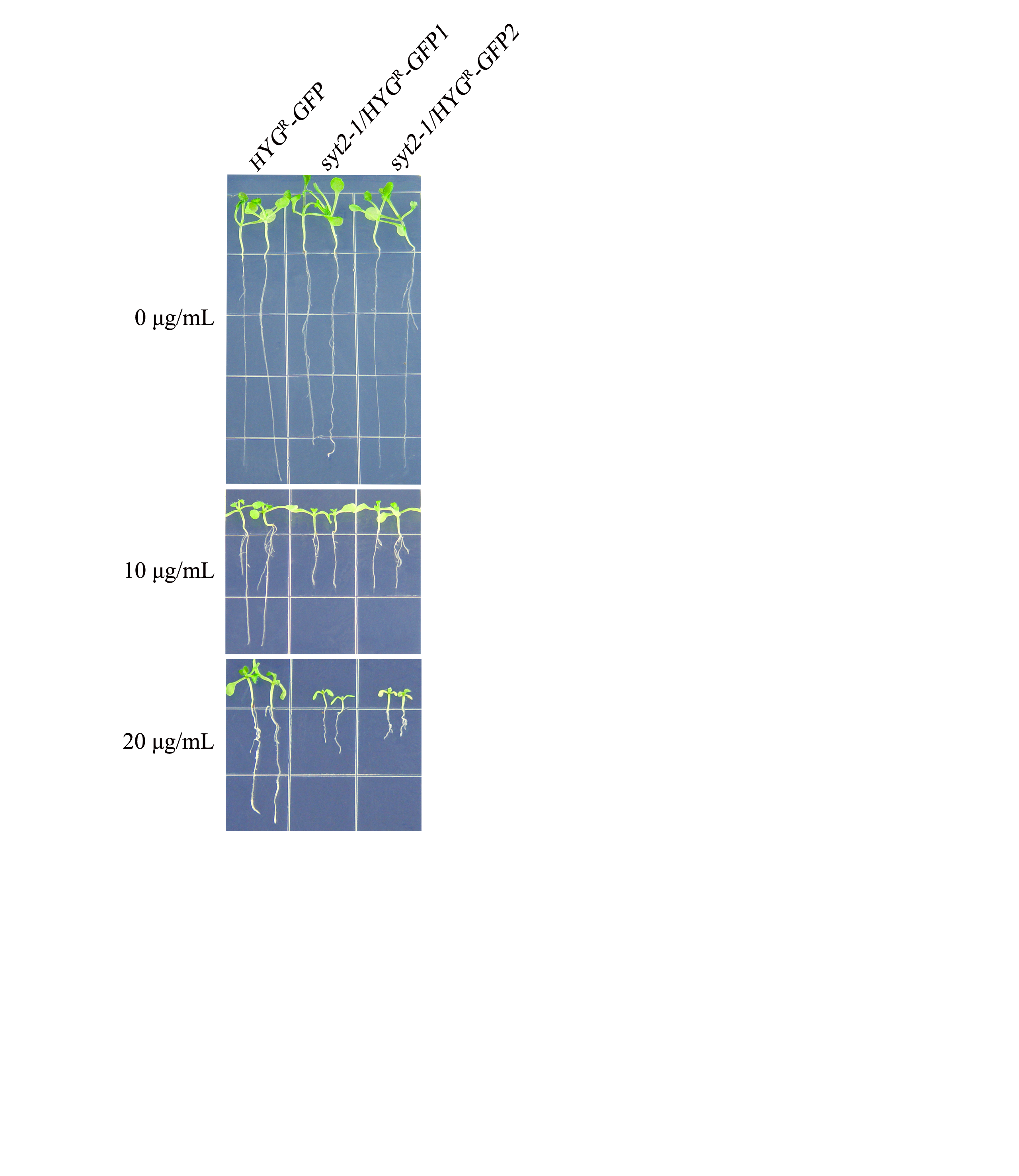

Supplement: Figure S6 — Responses of HYGR-GFP and syt2-1/HYGR-GFP seedlings to hygromycin B treatments. Seeds were germinated on ½ MS medium containing 0, 10 and 20 µg/mL hygromycin B and grown for 10 days before the pictures were taken. syt2-1/HYGR-GFP1 and syt2-1/HYGR-GFP2 were different lines that HYGR-GFP is expressed in SYT2 knock-out plants. (TIF) [file pone.0026477.s007.tif]

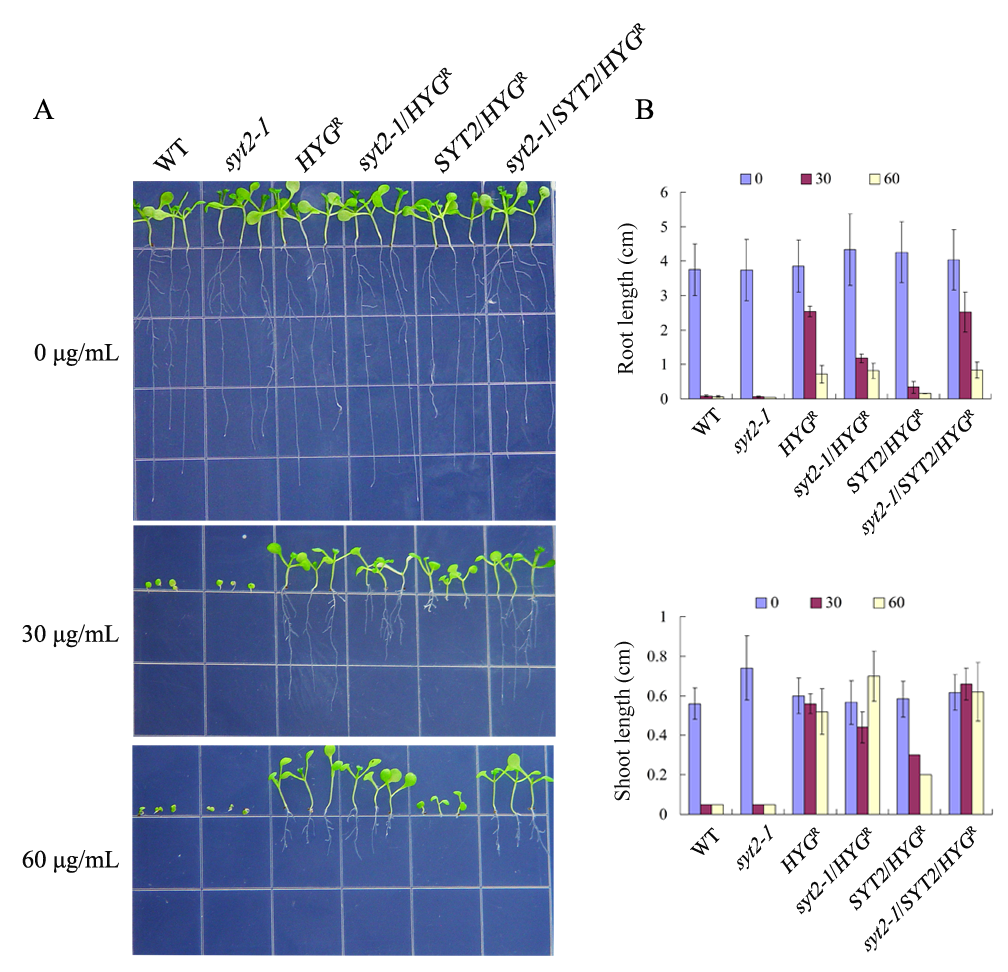

Supplement: Figure S7 — Expression of SYT2 complements the syt2-1 phenotype under hygromycin B treatment. (A) Seeds were germinated on ½ MS medium containing 0, 30 and 60 µg/mL hygromycin B and grown for 10 days before the pictures were taken. (B) Measurement of length of roots and shoots for seedlings treated as described for (A). Values are the means ± SD of 30–40 seedlings from three independent experiments. (TIF) [file pone.0026477.s008.tif]
